# Altered insulin secretion dynamics relate to oxidative stress and inflammasome activation in children with obesity and insulin resistance

**DOI:** 10.1186/s12967-023-04337-7

**Published:** 2023-08-20

**Authors:** Álvaro González-Domínguez, Thalía Belmonte, Jesús Domínguez-Riscart, Pablo Ruiz-Ocaña, Inés Muela-Zarzuela, Ana Saez-Benito, Raúl Montañez-Martínez, Rosa M. Mateos, Alfonso M. Lechuga-Sancho

**Affiliations:** 1 Grupo de Inflamación, Nutrición, Metabolismo y estrés Oxidativo, Instituto de Investigación e Innovación Biomédica de Cádiz (INiBICA), c/Doctor Marañón, 3 - Edificio Andrés Segovia, 11002 Cádiz, Spain; 2 Translational Research in Respiratory Medicine, University Hospital Arnau de Vilanova and Santa Maria, IRBLleida, Lleida, Spain; 3 CIBER of Respiratory Diseases (CIBERES), Institute of Health Carlos III, Madrid, Spain; 4 Unidad de Endocrinología Pediátrica y Diabetes, Servicio de Pediatría, Hospital Universitario Puerta del Mar, Cádiz, Spain; 5 Unidad de Endocrinología Pediátrica y Diabetes, Servicio de Pediatría, Hospital Universitario de Jerez, Jerez de la Frontera, Spain; 6 Grupo de Inflamación y Metabolismo Durante el Envejecimiento, Instituto de Investigación e Innovación Biomédica de Cádiz (INiBICA), Cádiz, Spain; 7 Servicio de Análisis Clínicos, Hospital Universitario Puerta del Mar, Cádiz, Spain; 8 Grupo de Diabetes Mellitus - Autoinmunidad y complicaciones crónicas, Implicaciones Patológicas, clínicas y terapéuticas, Instituto de Investigación e Innovación Biomédica de Cádiz (INiBICA), Cádiz, Spain; 9 Grupo de Daño cerebral Perinatal, Instituto de Investigación e Innovación Biomédica de Cádiz (INiBICA), Cádiz, Spain; 10 Departamento de Biomedicina, Facultad de Ciencias, Biotecnología y Salud Pública y Salud Pública, Universidad de Cádiz, Puerto Real, Spain; 11 Departamento Materno Infantil y Radiología, Facultad de Medicina, Universidad de Cádiz, Cádiz, Spain

**Keywords:** Childhood obesity, Inflammasome activation, Insulin resistance, Oral glucose tolerance test, Oxidative stress

## Abstract

**Background:**

Insulin resistance (IR) is considered the main driver of obesity related metabolic complications, and is related to oxidative stress and inflammation, which in turn promote each other. There is currently no specific definition of IR in children, rather, that for adult population is used by pediatric endocrinologists instead. Altered insulin secretion dynamics are associated with worse metabolic profiles and type 2 diabetes mellitus development, thus we aimed to test whether insulin response relates to oxidative stress and inflammation in children.

**Methods:**

We conducted a case–control study, including 132 children classified as follows: 33 children without obesity (Lean); 42 with obesity but no IR according to the American Diabetes Association criteria for adults (OBIR-); 25 with obesity and IR and an early insulin response to an oral glucose tolerance test (OGTT) (EP-OBIR +); 32 with obesity, IR, and a late insulin peak (LP-OBIR +); and studied variables associated with lipid and carbohydrate metabolism, oxidative stress, inflammation and inflammasome activation.

**Results:**

The measured parameters of children with obesity, IR, and an early insulin response were similar to those of children with obesity but without IR. It was late responders who presented an impaired antioxidant system and elevated oxidative damage in erythrocytes and plasma, and inflammasome activation at their white blood cells, despite lower classical inflammation markers. Increased uric acid levels seems to be one of the underlying mechanisms for inflammasome activation.

**Conclusions:**

It is insulin response to an OGTT that identifies children with obesity suffering oxidative stress and inflammasome activation more specifically. Uric acid could be mediating this pathological inflammatory response by activating NLRP3 in peripheral blood mononuclear cells.

**Graphical Abstract:**

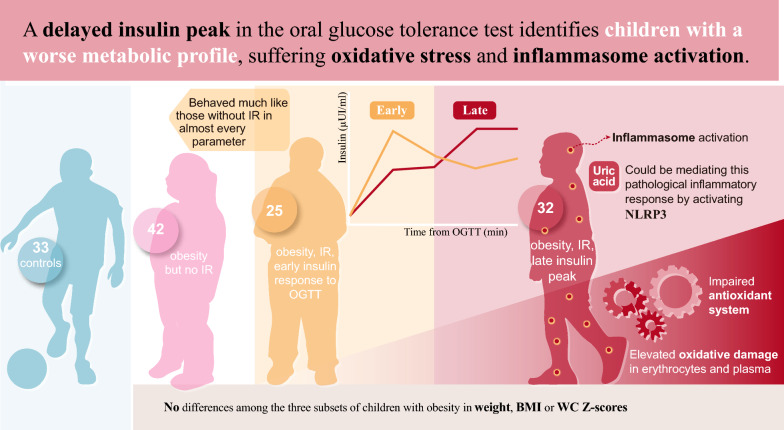

**Supplementary Information:**

The online version contains supplementary material available at 10.1186/s12967-023-04337-7.

## Highlights

- The definition of Insulin Resistance in children with obesity remains controversial, and we still use criteria defined in adults.
- A delayed insulin peak in the oral glucose tolerance test identifies children with a worse metabolic profile, suffering oxidative stress and inflammasome activation.
- This worse metabolic profile, oxidative stress and inflammasome activation are not well identified by classical inflammatory markers or indices.
- Uric acid is increased in children with a delayed insulin peak, and activates inflammasome, suggesting it may be playing a role in the development of obesity related complications.

**Supplementary Information:**

The online version contains supplementary material available at 10.1186/s12967-023-04337-7.

## Introduction

Childhood obesity is considered a main driver of detrimental health [1]. It is linked to the development of a cluster of metabolic complications known as Metabolic Syndrome (MS). Insulin resistance (IR) has been attributed a pivotal role in MS development, and may already be present in children with obesity as young as under 5 years of age [2, 3].

Insulin secretion in response to nutrient intake occurs physiologically in two phases: a rapid but short first one (with a higher effectivity in glucose levels reduction), followed by a more sustained second phase. In adults, attenuation of the first phase of insulin secretion is associated with impaired glucose tolerance [4, 5]. In children with obesity, differences in insulin curve morphology along oral glucose tolerance test (OGTT) also relate to metabolic complications. Children with a late insulin response, exhibit lower levels of high-density lipoprotein cholesterol (HDL-C), higher ratio triglycerides/HDL-C, higher uric acid levels and a higher homeostatic model assessment of insulin resistance (HOMA-IR) [6].

Adipose tissue expansion and macrophage infiltration in people with and animal models of obesity lead to low-grade chronic inflammation which triggers IR. These activated adipose tissue macrophages secrete proinflammatory cytokines which serve as chemotactic signals contributing to an increased macrophage infiltration, adipose cells dysfunction, and systemic IR via paracrine effects on surrounding tissue. Thus, once initiated, IR and inflammation amplify each other [7–9]. In children with obesity, levels of peripheral blood neutrophils and monocytes are increased and correlate with IR [10, 11]. These innate immune cells detect danger-associated molecular patterns (DAMPs) and mediate NOD like receptor 3 (NLRP3) activation in a two-step process triggered by IL-1α, tumor necrosis factor α, or uric acid among others [12, 13]. NLRP3 interacts with procaspase-1, establishing the NLRP3 inflammasome and activating caspase-1, which in turn mediates IL-1β and IL-18 maturation and secretion. Since nutrient excess generates DAMPs, NLRP3 activation has been linked to chronic inflammation in obesity and IR [14]. Moreover, macrophages produce IL-1β in a glucose-dependent manner, promoting insulin action and inflammation [15].

Inflammation and oxidative stress (OS) are tightly interrelated in a vicious cycle. Activated immune cells release reactive oxygen species (ROS) which if maintained, may lead to exacerbated OS. Indeed, ROS prime signaling cascades that promote proinflammatory gene expression [16, 17]. Additionally, under a pro-oxidative environment, lipids and proteins are modified and may act as DAMPs, triggering innate immune responses as mentioned before [18].

Given the strong relationship between glucose metabolism, inflammation and OS, we hypothesized that children with obesity and a late insulin peak at the OGTT, may also have increased OS levels and inflammation.

## Subjects and methods

We performed an observational case–control study, involving children with obesity (BMI >  + 2 SD for age and sex) who,for clinical reasons, required an OGTT to assess their carbohydrate metabolism. Recruitment was consecutive at the outpatient clinic of the Pediatric Endocrinology Unit of a single University Hospital in Cádiz, Spain. Every OGTT was performed after overnight fasting, with 75 gr of sucrose (GlycoSull, QCA S.A., Tarragona, Spain). IR was diagnosed according to the ADA criteria: a HOMA-IR above 3.5, fasting insulin > 15 µUI/mL, insulin at 120 of OGTT > 75 µUI/mL or insulin at any time-point of the curve > 150 µUI/mL [19, 20]. The group of children with obesity was later divided into three according to their carbohydrate status: a group with no evidence of IR (OBIR-), a second group with obesity and IR (OBIR +), presenting the maximum insulin peak in the first 30 min of the OGTT, considered as “early peak” (EP-OBIR +), and a third group with obesity and IR, presenting the maximum insulin concentration at minutes 90 or 120 of the OGTT, considered as “late peak” (LP-OBIR +). Lean childr participants required blood tests for other non-acute medical conditions (i.e. pre-anesthesia evaluation). The age of every participant included in the study ranged from 8 to 12 years. Sample size was calculated using GRANMO 7.12 open software, based on previous data on lipid peroxidation levels in children with obesity and IR [21]. Accepting an alpha risk of 0.05 and a beta risk of 0.2, assuming a common standard deviation of 0.47, the estimated sample size to recognize as statistically significant a difference greater or equal to 0.5 units, is 15 subjects per group, for the comparison between groups. For intragroup comparisons and accepting the same risks in a two-sided test, 10 subjects per group were calculated aiming for a difference greater than 0.5, assuming a common standard deviation of 0.5 and a correlation coefficient between initial and final measurements of 0.8. The study was carried out in accordance with The Code of Ethics of the World Medical Association (Declaration of Helsinki) for experiments involving humans and it was approved by the Human Ethics Committee of our Institution. Written informed consent was obtained from each participant/parent/legal guardian.

### Sample collection and preparation

Additional venous blood samples were extracted in serum (gelose) and plasma (EDTA) separating tubes from every child after fasting overnight (T0), and at 60 and 120 min during the OGTT. Tubes were centrifuged at 1500 g for 10 min at 4 ºC to obtain serum and plasma samples. Plasma-separating tubes were also used for obtaining erythrocytes for OS and antioxidant biomarker determination. Briefly, erythrocytes were washed 3 times with cold saline solution (NaCl 9 g/L) by centrifuging for 5 min at 1500 g and 4 ºC. Peripheral blood mononuclear cells (PBMCs) were isolated from 10 mL EDTA tubes by density gradient over Hystopaque-1077 (Sigma-Aldrich, St. Louis, MO) following standard procedures. Every fraction was immediately frozen at -80ºC to ensure its correct preservation.

### Anthropometry and biochemical analysis of the study population

Demographic data, personal and familiar cardiovascular antecedents and risk factors were collected at medical interview. Anthropometric data were measured and puberal findings were evaluated by pediatric endocrinologists. Z-scores for anthropometric variables were calculated, using Spanish reference values [22].

Biochemical analysis included insulin and glucose determination at basal and at different times along the OGTT, as well as a complete blood cell count and inflammatory markers such as uric acid, ferritin, creatinine, or C-reactive protein (CRP). The HOMA-IR was calculated with the formula: HOMA-IR = [(fasting insulin (mIU/L) x fasting glucose (mg/dl) × 0.055/22.5]. The areas under the curve for glucose and insulin (AUCg and AUCi) were calculated applying the formula: AUCg/i = 0.25 × Glucose/insulin 0’ + 0.5 × Glucose/insulin 30’ + 0.75 × Glucose/insulin 60’ + 0.5 × Glucose/insulin 120’. Castelli index was calculated dividing total cholesterol by the HDL-C. Biochemical analysis were performed in serum by using standard clinical assays at the Clinical Analysis Department of our Hospital. Insulin and ferritin were measured in the Abbott Alinity i analyzer, and glucose, creatinine, CRP, and uric acid were measured in the Abbott Alinity c analyzer, using Alinity i and c reagent Kits (Abbott Molecular, Des Plaines, Il).

### Oxidative stress markers

#### Lipid peroxidation quantification

The formation of thiobarbituric acid-reacting substances (TBARS) was measured by the method described by *Buege *et al*.* [23]. Briefly, 100 µL of diluted samples (1:1, v/v; in water) were mixed with 400 µL of the reaction solution, composed of 2.5 N HCl, 0.375% (w/v) thiobarbituric acid, 15% (w/v) trichloroacetic acid, and 0.01% (w/v) butylated hidroxytholuene. The mix was then heated at 100 ºC for 15 min and centrifuged at 900 g for 5 min. The malondialdehyde (MDA) standard curve was prepared in the same way but without heating and centrifuging. Finally, the standard curve and 200 µL of each sample were added to a 96-well microplate and the absorbance was recorded at 535 nm. Results were expressed as nmol of MDA per mg of proteins.

#### Carbonyl groups determination

Carbonyl groups (C = O) were determined following the method described by *Levine *et al*.* [24]. To this end, proteins were purified from samples and incubated with 2,4-dinitrophenylhydrazine (DNPH) dissolved in 2.5 N HCl (Sigma-Aldrich, Saint Louis, USA). After reacting with carbonyl groups, DNPH is converted into 2,4-phenylhydrazone which, in the presence of 6 M HCl-guanidine, can be determined spectrophotometrically at 370 nm. Absorbance at 280 nm was measured in blanks without DNPH to estimate the protein content of the samples using a BSA standard curve prepared in 6 M HCl-guanidine. Results were expressed as nmol of C = O per mg of proteins in erythrocytes.

#### Cytosolic ROS quantification

Cytosolic ROS were evaluated using 2,7-dichlorofluorescein diacetate (DCFDA) (Abcam, Cambridge, United Kingdom). Briefly, 20 µL of erythrocytes from blood samples collected in plasma-separating tubes after centrifugation, were either incubated with the same volume of RPMI medium (Fisher Scientific, Leicestershire, UK) as negative control or DCFDA 60 μM for 30 min at 37 ºC and in darkness. Then, cells were washed with PBS1X and fluorescence was measured at 485 nm excitation and 528 nm emission using a FACS-Calibur cytometer and the CellQuest Pro software (Becton Dickinson; San Jose, CA, USA).

#### Osmotic fragility

Fresh blood (20 µL) was immediately mixed with different saline solutions of increasing concentration and incubated for 30 min at room temperature. Samples were then centrifuged at 1500 g for 10 min, and the supernatant hemoglobin content was estimated by recording its absorbance at 540 nm.

### Antioxidant capacity

#### Total antioxidant capacity

Total antioxidant capacity (TAC) was estimated following the method described by *Erel *et al*.* [25], based on the competition between o-dianosidine and antioxidant agents for hydroxyl radicals present in the sample. The higher the antioxidant agent concentration is, the lesser the reaction with o-dianosidine molecules is, with a subsequent reduction in absorbance changes. Samples were mixed with a 10 mM o-dianisidine dihydrochloride solution and basal absorbance was recorded at 444 nm. After the addition of H_2_O_2_ 75 mM, samples were incubated for 4 min, and absorbance was recorded again. The difference between both values excludes endogenous H_2_O_2_ conversion and is representative of the amount of antioxidant agents in the sample. Results were expressed as μmol of Trolox equivalents per mg of proteins.

#### Glutathione reductase activity

As described by *Edwards *et al*.*, glutathione reductase (GR) activity was determined following the NADPH oxidation rate [26]. A buffer consisting of 0.1 M HEPES–NaOH pH 7.8, 1 mM EDTA and 3 mM MgCl_2_ was added to a 96-well microplate either with 0.5 mM oxidized glutathione (samples) or without it (blanks). Then, 5 µL of sample were added together with 0.2 mM NADPH to start the reaction. Absorbance at 340 nm was monitored for 2 min with determinations each 10 s. The molar extinction coefficient for NADPH is 6.22 mM^−1^ cm^−1^. The enzymatic activity was expressed as nmol NADP min^−1^ per mg of protein.

#### NADP-dehydrogenase activities

Both glucose-6-phosphate and 6-phosphogluconate dehydrogenases (G6PDH and 6PGDH) activities were determined spectrophotometrically by quantifying the NADP^+^ reduction rate as described by *Mateos *et al*.* [27]. Briefly, 10 μL of sample were mixed with 36.11 mM HEPES and 0.8 mM NADP^+^. Finally, substrate (glucose-6-phosphate for G6PDH activity and 6-phosphogluconate for 6PGDH activity) was added to trigger the reaction. Then, absorbance was followed at 340 nm for 3 min with determinations every 20 s. The molar extinction coefficient for NADP^+^ was 6.22 mM-1 cm-1. The enzymatic activity was expressed as nmol NADP min-1 per mg of protein.

### Inflammatory ratios

Neutrophil, leukocyte, platelet, lymphocyte and monocyte absolute counts were used to calculate the systemic immune-inflammation index (SII = neutrophils × platelets/lymphocytes), systemic inflammation response index (SIRI = neutrophils × monocytes/lymphocytes), aggregate index of systemic inflammation (AISI = neutrophils × monocytes × platelets/lymphocytes), monocyte to lymphocyte ratio (MLR = monocytes/lymphocytes), and neutrophil to lymphocyte ratio (NLR = neutrophils/lymphocytes).

### Western blotting

PBMCs cellular lysates were prepared by gentle shaking and applying 10 pulses of sonication (Branson Sonifier 150) in RIPA buffer supplemented with protease inhibitor PhenylMethylSulfonylFluoride 1 mM. Similarly, plasma samples were diluted in RIPA buffer (1:1, v/v) and sonicated as previously described. Protein content was determined using the Bradford method and electrophoresis was carried out in 12% acrylamide SDS–PAGE, loading 60 µg samples. Then, proteins were transferred to PVDF membranes (Biorad, Hercules, CA, USA) and incubated with primary antibody solutions [1:1000] for NLRP3 (D4D8T, Cell Signaling), IL-1β (D2F3B, Cell Signaling), caspase-1 (2225, Cell Signaling), and gasdermin-D (sc-376318, Santa Cruz) quantification. Membranes were then incubated with their respective secondary antibody [1:2000] and immunolabeled proteins were detected by chemiluminescence method using Western Bright Sirius HRP substrate (Advansta, San Jose, CA, USA). Western blot images were finally quantified using Image-Lab software (Biorad, Hercules, CA, USA).

### Statistical analysis

Data analyses were conducted using GraphPad Prism 8. Data normality was assessed using the Kolmogorov–Smirnov test. For parametric variables, the t-test was used for pairwise comparisons, and ANOVA test with Bonferroni post hoc corrections was used for comparisons of more than two groups. For non-parametric variables, Mann Whitney U test was used for pairwise comparisons, and Kruskal–Wallis test with Dunn’s post hoc corrections was used for comparisons of more than two groups. Correlations between variables were assessed using the Spearman’s test. P-values under 0.05 were considered significant.

## Results

We included a total of 132 children (56 girls) in the study. Of these, 33 were lean controls and the remaining 99 had obesity. Of the latter, 42 were OBIR-, 25 had an early insulin peak (EP-OBIR +), and 32 showed a late insulin peak (LP-OBIR +).

Children in the different groups had similar ages and sex distribution. All three groups with obesity were taller and heavier than children in the control group. Of note, there were no differences among the three subsets of children with obesity in weight, BMI or WC Z-scores. LP-OBIR + children were taller than OBIR-. This same difference was seen in systolic and diastolic blood pressures (BP) (mmHg), but not in BPs Z-score. Regarding neonatal antecedents, we found small but significant differences between groups in their gestational age. EP-OBIR + were born earlier than both, OBIR- and LP-OBIR + . However, the clinical relevance of this finding is unlikely given they were all born at term. Anthropometrical parameters are summarized in Table 1.

**Table 1:** Anthropometric characteristics of the study population

	Lean	OBIR-	EP-OBIR +	LP-OBIR +
Number of subjects (N)	33	42	25	32
Sex (% male)	60.60	42.86	64.00	68.75
Age (years)	10.34 ± 0.29	10.34 ± 0.38	10.94 ± 0.47	10.84 ± 0.25
WC (cm)	60.07 ± 0.66	93.91 ± 2.48^*^	95.33 ± 1.93^*^	101.78 ± 1.91^*,a,b^
WC (Z-Score)	− 0.51 ± 0.09	4.70 ± 0.33^*^	4.85 ± 0.39^*^	4.93 ± 0.23^*^
Weight (kg)	31.34 ± 0.70	61.87 ± 2.73^*^	64.54 ± 2.85^*^	70.19 ± 1.93^*,b^
Weight (Z-Score)	− 0.15 ± 0.12	4.36 ± 0.21^*^	5.02 ± 0.36^*^	5.02 ± 0.22^*^
Height (cm)	135.41 ± 1.37	145.24 ± 2.02^*^	151.64 ± 2.13^*^	151.38 ± 1.83^*^
Height (Z-Score)	− 0.33 ± 0.17	0.98 ± 0.13^*^	1.48 ± 0.27^*^	1.79 ± 0.14^*,b^
BMI (Kg /m2)	16.55 ± 0.12	28.67 ± 0.56^*^	29.96 ± 0.67^*^	30.46 ± 0.70^*^
BMI (Z-Score)	− 0.56 ± 0.10	4.14 ± 0.17^*^	4.28 ± 0.17^*^	4.50 ± 0.27^*^
Systolic BP (mmHg)	102.71 ± 1.38	113.47 ± 1.57^*^	118.81 ± 2.14^*^	121.6 ± 1.80^*,b^
Systolic BP (Z-Score)	0.07 ± 0.10	0.99 ± 0.14^*^	1.13 ± 0.17^*^	1.41 ± 0.18^*^
Diastolic BP (mmHg)	65.96 ± 1.05	73.13 ± 1.14^*^	75.82 ± 1.16^*^	77.07 ± 1.02^*,b^
Diastolic BP (Z-Score)	0.39 ± 0.10	1.00 ± 0.09^*^	1.12 ± 0.09^*^	1.24 ± 0.10^*^
Gestational age (weeks)	39.64 ± 0.26	40.00 ± 0.18	38.78 ± 0.36^b^	40.04 ± 0.32^a^
Newborn height (cm)	50.69 ± 0.46	50.54 ± 0.34	50.06 ± 0.86	48.95 ± 0.86
Newborn height (Z-Score)	0.39 ± 0.23	0.71 ± 0.24	0.77 ± 0.25	0.58 ± 0.34
Newborn weight (g)	3287.31 ± 92.12	3361.97 ± 61.32	3048.33 ± 128.82^b^	3143.64 ± 106.90
Newborn weight (Z-Score)	− 0.18 ± 0.20	0.35 ± 0.17^*^	− 0.08 ± 0.30	0.33 ± 0.21

Values are shown as the mean ± SEM. (*) indicates difference with respect to lean children, (b) indicates difference with respect to OBIR- children, and (a) indicates difference with respect to EP-OBIR + children

ObIR-, non-insulin resistant children with obesity; ObIR + , insulin resistant children with obesity; EP-OBIR + , early peak OBIR + children; LP-OBIR + , late peak OBIR + children; WC, waist circumference; BMI, body mass index; BP, blood pressure

Both OBIR + groups had similar mean glucose and mean insulin along the curve and both were higher than in the OBIR- group. Additionally, the LP-OBIR + group had higher glucose levels at the end of the curve. When analyzing OGTT results, the LP-OBIR + group presented higher glucose levels at the end of the curve. All of the groups with obesity showed a more unfavorable lipidic profile than controls, reflected by higher triglyceride levels, higher Castelli index, and lower HDL-C levels. The LP-OBIR + group showed even lower levels of HDL-C and LDL-C when compared to OBIR- children, and tended to have higher triglycerides levels and Castelli index. Every group had normal GOT and GPT transaminases levels (Table 2).

**Table 2:** Biochemical characteristics of the study population

	Lean	OBIR-	EP-OBIR +	LP-OBIR +
Blood glucose curve (mg/dL)				
0 min	84.07 ± 0.77	83.19 ± 0.62	86.59 ± 1.56	85.13 ± 1.46
30 min		140.43 ± 6.35	146.50 ± 3.53	134.60 ± 7.23
60 min		116.30 ± 3.69	129.87 ± 3.87^b^	129.55 ± 4.06^b^
90 min		105.78 ± 2.11	119.13 ± 2.65^b^	130.52 ± 4.50^b^
120 min		104.23 ± 2.57	120.05 ± 2.33^b^	133.85 ± 2.95^a,b^
Blood insulin curve (µUI/mL)				
0 min	5.42 ± 0.36	11.45 ± 0.53^*^	21.32 ± 1.41^*,b^	21.00 ± 1.48^*,b^
30 min		60.33 ± 5.58	162.39 ± 10.72^b^	97.33 ± 6.02^a,b^
60 min		67.57 ± 5.28	121.84 ± 8.71^b^	91.65 ± 6.71^a,b^
90 min		56.97 ± 4.08	99.06 ± 8.17^b^	114.48 ± 7.76^b^
120 min		43.62 ± 3.11	116.61 ± 1.99^b^	137.11 ± 6.66^a,b^
HOMA-IR (A.U.)	1.09 ± 0.07	2.43 ± 0.10^*^	4.09 ± 0.30^*,b^	4.47 ± 0.35^*,b^
Mean glucose (mg/dL)		105.38 ± 1.92	118.13 ± 2.57^b^	120.58 ± 2.72^b^
Mean insulin (µUI/mL)		48.88 ± 2.46	111.80 ± 7.39^b^	123.40 ± 9.38^b^
AUCg (mg·h/dL)		198.76 ± 8.20	231.52 ± 9.69^b^	230.81 ± 7.99^b^
AUCi (µU·h/mL)		106.78 ± 6.75	257.46 ± 14.56^b^	198.24 ± 10.73^a,b^
Total Cholesterol (mg/dL)	173.48 ± 4.74	157.95 ± 3.46^*^	157.71 ± 5.35	148.54 ± 3.26^*^
Triglycerides (mg/dL)	45.38 ± 2.17	64.15 ± 3.24^*^	65.14 ± 5.49^*^	70.68 ± 2.44^*^
HDL-C (mg/dL)	60.65 ± 1.42	46.26 ± 0.83^*^	44.00 ± 0.78^*^	41.88 ± 1.26^*,b^
LDL-C (mg/dL)	98.29 ± 2.84	96.11 ± 3.01	95.08 ± 4.58	87.33 ± 2.73^*,b^
Castelli Index (A.U.)	2.78 ± 0.10	3.32 ± 0.10^*^	3.38 ± 0.12^*^	3.55 ± 0.13^*^
GOT (U/L)	25.88 ± 0.53	22.29 ± 0.76^*^	19.19 ± 0.44^*,b^	21.00 ± 0.56^*,a^
GPT (U/L)	16.29 ± 0.73	17.89 ± 0.66	21.05 ± 1.21^*^	19.91 ± 0.92^*^

Values are shown as the mean ± SEM. (*) indicates difference with respect to lean children, (b) indicates difference with respect to OBIR- children, and (a) indicates difference with respect to EP-OBIR + children

ObIR-, non-insulin resistant children with obesity; ObIR + , insulin resistant children with obesity; EP-OBIR + , early peak OBIR + children; LP-OBIR + , late peak OBIR + children; HOMA-IR, homeostasis model assessment of insulin resistance; A.U., arbitrary units; AUCg, area under the curve of glucose; AUCi, area under the curve of insulin; HDL-C, high density lipoprotein cholesterol; LDL-C, low density lipoprotein cholesterol; GOT, aspartate transaminase; GPT, alanine transaminase

### Oxidative stress markers

#### Reactive oxygen species production.

**Fig. 1:**
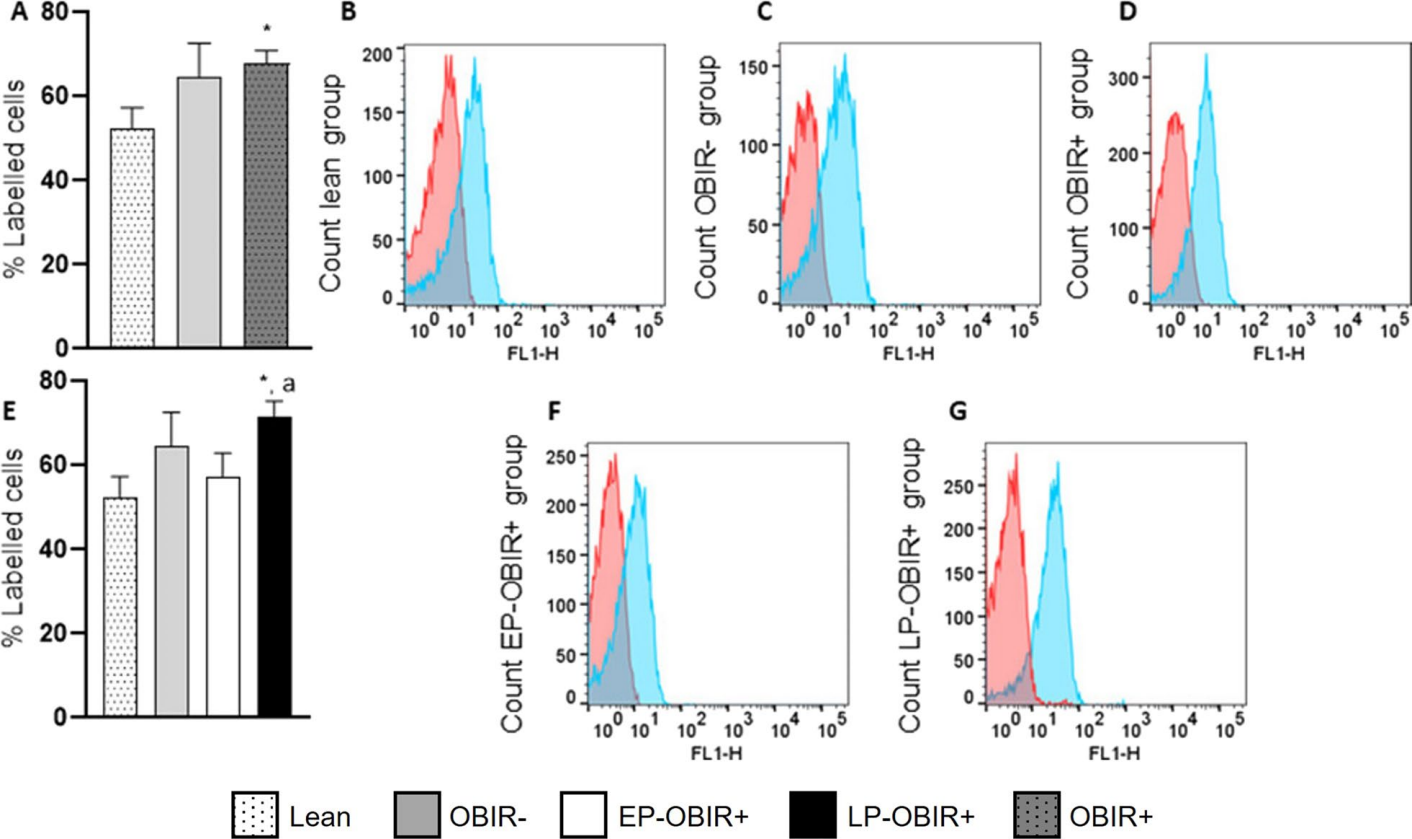
Levels of reactive oxygen species in erythrocytes in the study population. White bars with dots represent lean children (n = 15), grey bars represent OBIR- children (n = 10), grey bars with dots represent OBIR + children (n = 47), white bars represent EP-OBIR + children (n = 10), and black bars represent LP-OBIR + children (n = 26). **A** shows ROS levels in lean, OBIR- and OBIR+ groups of children. Representative histograms for lean, ObIR-, and ObIR + groups are represented in **B**, **C** and **D**, respectively (red represents negative control, and blue DCFDA labelled cells). **E** shows ROS levels in these same groups, when the ObIR + group is subdivided into early responders (white bars) and late responders (black bars). **F** and **G** are representative histograms for these early and late responders respectively. Values are means ± SEM. P < 0.05 was considered statistically significant. (*) shows significant differences relative to lean children and **a** shows significant differences relative to EP-OBIR + children. ObIR-, non-insulin resistant children with obesity; ObIR + , insulin resistant children with obesity; EP-OBIR + , early peak OBIR + children; LP-OBIR + , late peak OBIR + children; DCFDA, 2,7-dichlorofluorescein diacetate

Erythrocytes from the OBIR + group displayed a higher ROS production than lean controls. When separately analyzing the EP-OBIR + and LP-OBIR + groups, we found that the ROS production in the EP-OBIR + group was similar to that of the OBIR- group, while the LP-OBIR + group had increased ROS production compared to lean controls and to the EP-OBIR + group (Fig. 1).

#### Oxidative damage

**Fig. 2:**
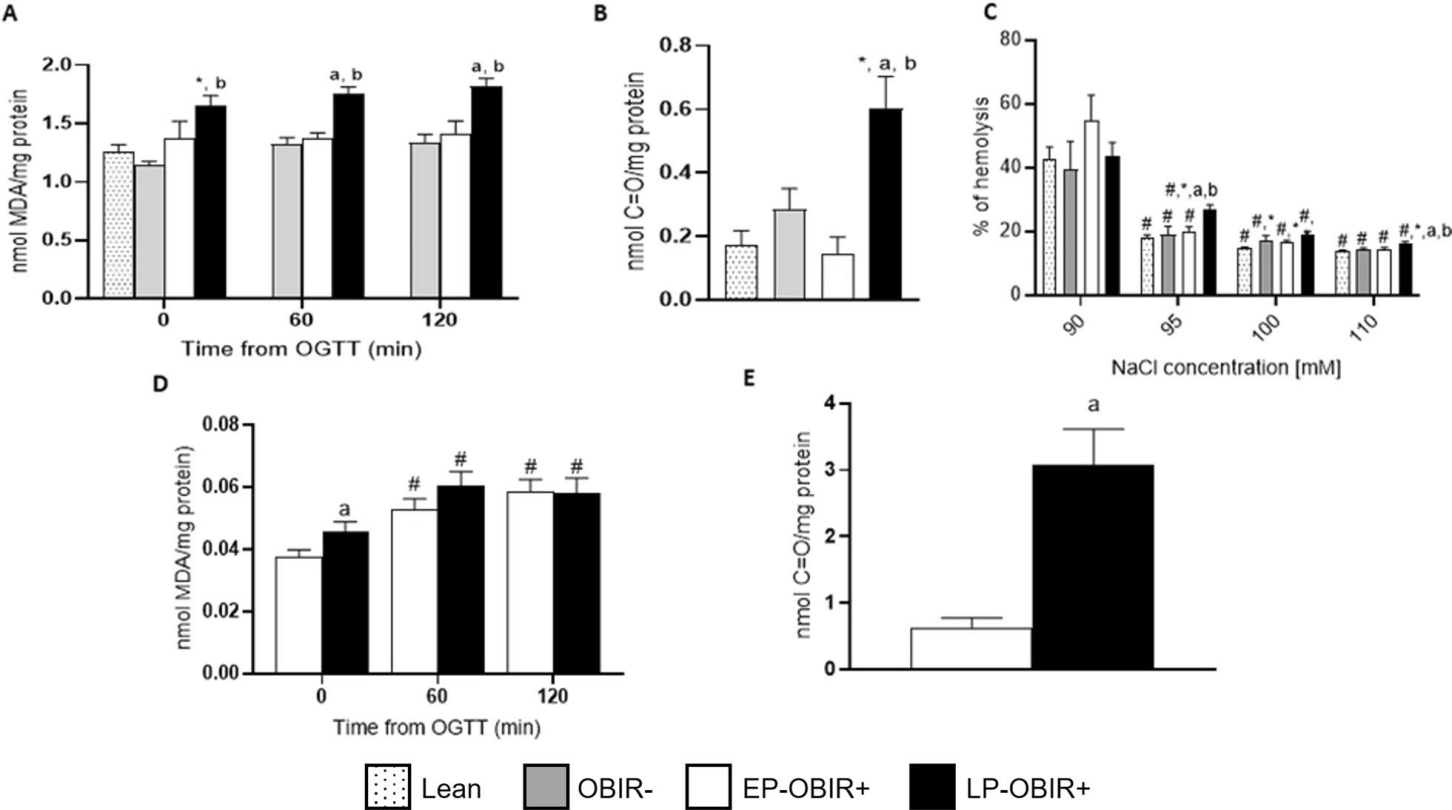
Levels of oxidative damage present in erythrocytes and plasma of the study population. TBARS levels were measured in erythrocytes **A** and plasma **D** at baseline and along OGTT, and carbonyl group levels were measured at baseline in erythrocytes **B** and plasma **E**. Also, erythroid osmotic fragility **C** was quantified in every group. White bars with dots represent lean children (n = 17), grey bars represent OBIR- children (n = 13), white bars represent EP-OBIR + children (n = 13), and black bars represent LP-OBIR + children (n = 17). Values are means ± SEM. P < 0.05 was considered for statistical significance. (#) shows significant differences relative to 90 mM of NaCl, (*) shows significant differences relative to lean children, **a** shows significant differences relative to EP-OBIR + children, and **b** shows significant differences relative to OBIR- children. ObIR-, non-insulin resistant children with obesity; ObIR + , insulin resistant children with obesity; EP-OBIR + , early peak OBIR + children; LP-OBIR + , late peak OBIR + children; OGTT, oral glucose tolerance test; MDA, malondialdehyde; TBARS, thiobarbituric acid reacting substances; C = O, carbonyl groups

Lipid peroxidation and protein oxidative damage was measured both in erythrocytes and plasma. Protein oxidative damage was measuredat baseline, and lipid peroxidation all along the OGTT curve. In erythrocytes, both parameters were increased at baseline in the LP-OBIR + group of children, and it only increased further along the OGTT in this same group (Fig. 2A, B). Additionally, osmotic fragility was higher in LP-OBIR + when compared to every other group even at low NaCl concentrations (Fig. 2C).

In plasma, a similar trend differentiating EP-OBIR + from LP-OBIR + groups was observed, but the levels of plasmatic TBARS increased along the curve in both OBIR + groups without differences between them (Figs. 2D, E).

### Antioxidant capacity

**Fig. 3:**
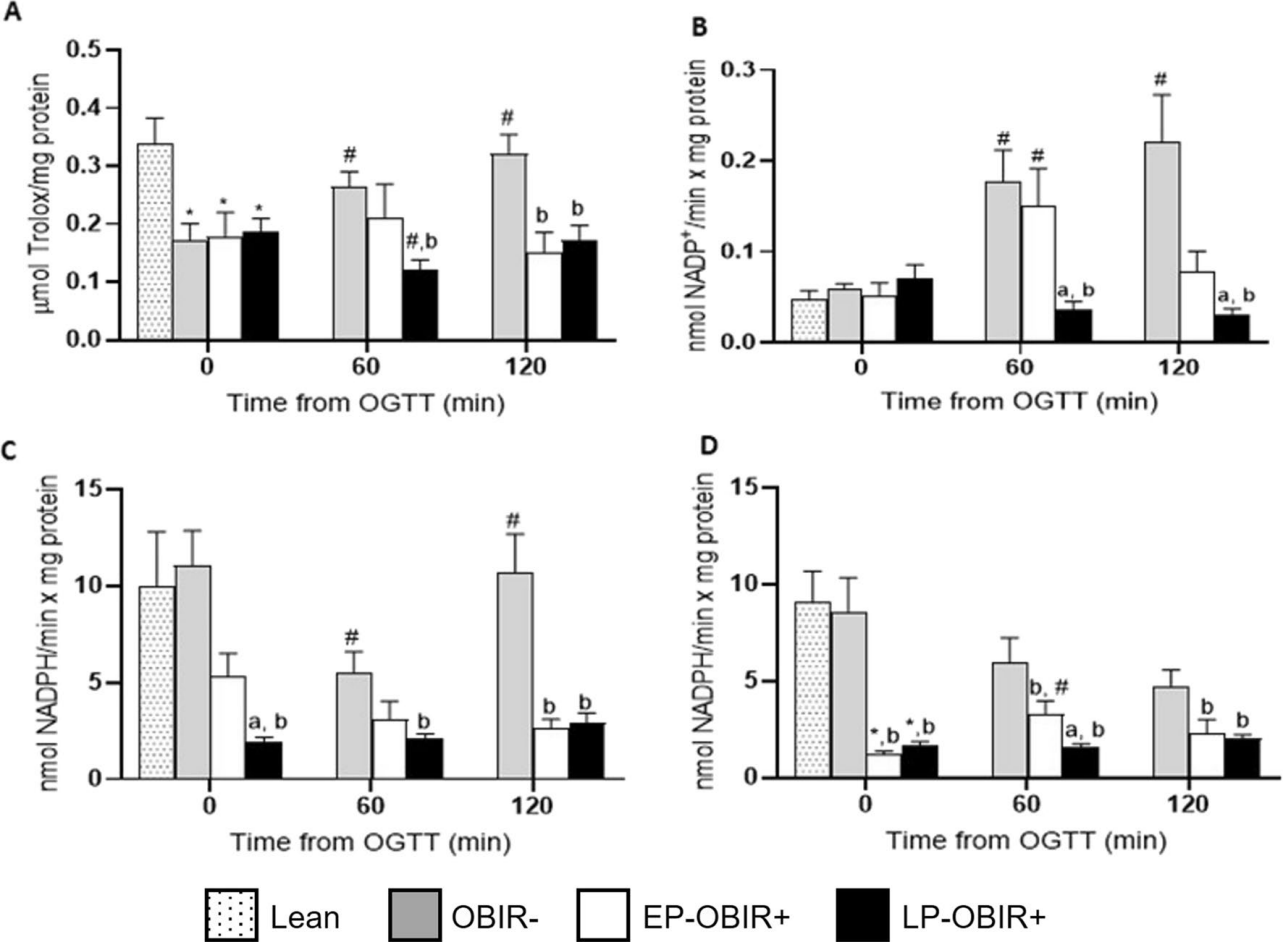
Antioxidant system-related enzyme activities in erythrocytes in the study population. TAC **A**, GR **B**, G6PDH **C**, and 6PGDH **D** activities were measured at baseline and along the OGTT. White bars with dots represent lean children (n = 20), grey bars represent OBIR- children (n = 13), white bars represent EP-OBIR + children (n = 10), and black bars represent LP-OBIR + children (n = 25). Values are means ± SEM. P < 0.05 was considered for statistical significance. (*) shows significant differences relative to lean children, **a** shows significant differences relative to EP-OBIR + children, **b** shows significant differences relative to OBIR- children, and (#) shows significant differences along the OGTT. ObIR-, non-insulin resistant children with obesity; ObIR + , insulin resistant children with obesity; EP-OBIR + , early peak OBIR + children; LP-OBIR + , late peak OBIR + children; NADP, nicotinamide-adenine dinucleotide phosphate; TAC, total antioxidant capacity; GR, glutathione reductase; G6PDH, glucose-6-phosphate dehydrogenase; 6PGDH, 6-phosphogluconate dehydrogenase; OGTT, oral glucose tolerance test

Children with obesity displayed approximately half of the TAC compared with controls at baseline (Fig. 3A). However, whereas the OBIR- group increased their TAC along the curve, the EP-OBIR + remained unchanged, and the LP-OBIR + group showed a decrease in TAC at the 60’ time point.

The four groups exhibited similar GR activity at baseline. While the OBIR- group increased this activity along the OGTT, the EP-OBIR + displayed a transient increase, and the LP-OBIR + group remained low (Fig. 3B). Finally, the OBIR- group and the lean control group showed similar G6PDH and 6PGDH basal activities, while both OBIR + groups had approximately 20% of these activities, and only the EP-OBIR + group increased its 6PGDH activity in a transient and mild manner its, while the OBIR- group showed a transient decrease in G6PDH activity along the OGTT (Figs. 3C, D).

### Red blood cell characterization

**Fig. 4:**
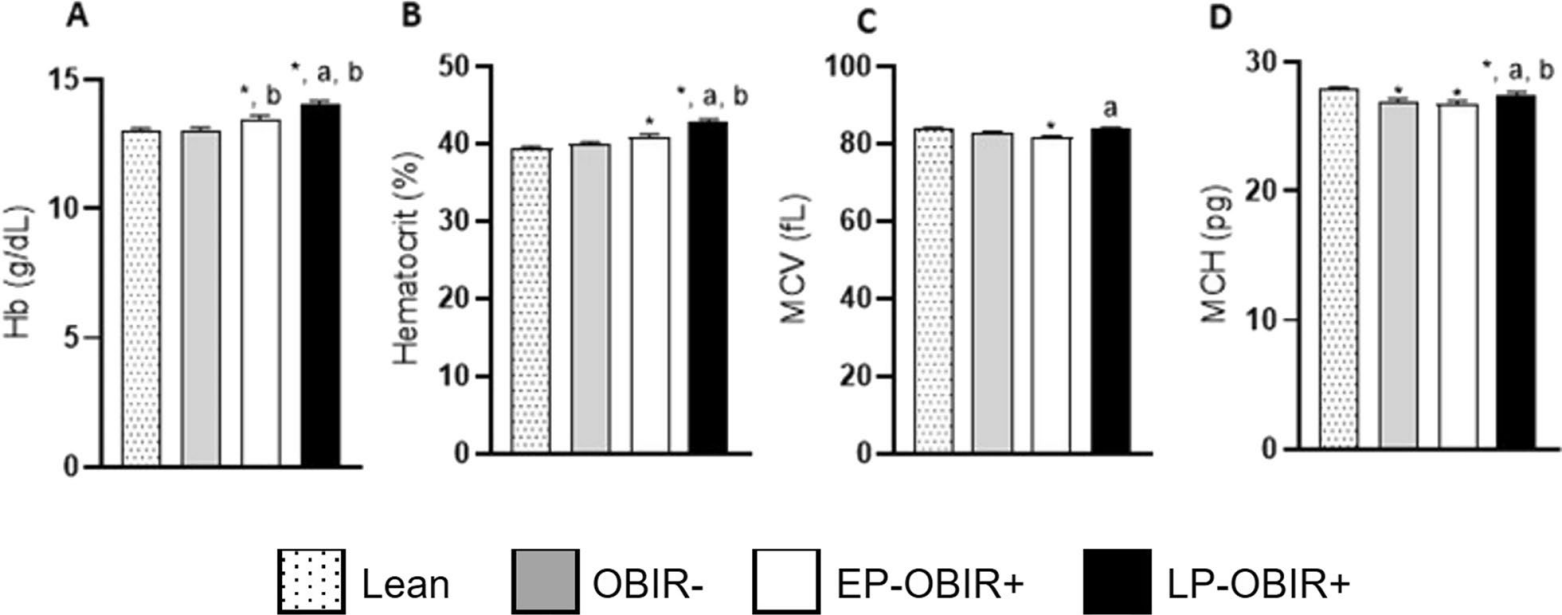
Erythrocyte characterization of the study population. Total hemoglobin content **A**, hematocrit **B**, mean corpuscular volume **C**, and mean corpuscular hemoglobin **D** were quantified in peripheral blood at baseline. White bars with dots represent lean children (n = 30), grey bars represent OBIR- children (n = 35), white bars represent EP-OBIR + children (n = 19), and black bars represent LP-OBIR + children (n = 32). Values are means ± SEM. P < 0.05 was considered for statistical significance. (*) shows significant differences relative to control children, **a** shows significant differences relative to EP-OBIR + children, and **b** shows significant differences relative to OBIR- children. ObIR-, non-insulin resistant children with obesity; ObIR + , insulin resistant children with obesity; EP-OBIR + , early peak OBIR + children; LP-OBIR + , late peak OBIR + children; Hb, hemoglobin; MCV, mean corpuscular volume; MCH, mean corpuscular hemoglobin

All the parameters were within normal values for sex and age. There were no children with anemia or other pathological conditions. Both OBIR + groups had higher levels of hemoglobin (Hb) ad a higher hematocrit, and the LP-OBIR + group had the highest of all (Fig. 4A, B). All three groups of children with obesity had lower mean corpuscular hemoglobin (MCH), (Fig. 4D).

### Inflammatory markers

**Fig. 5:**
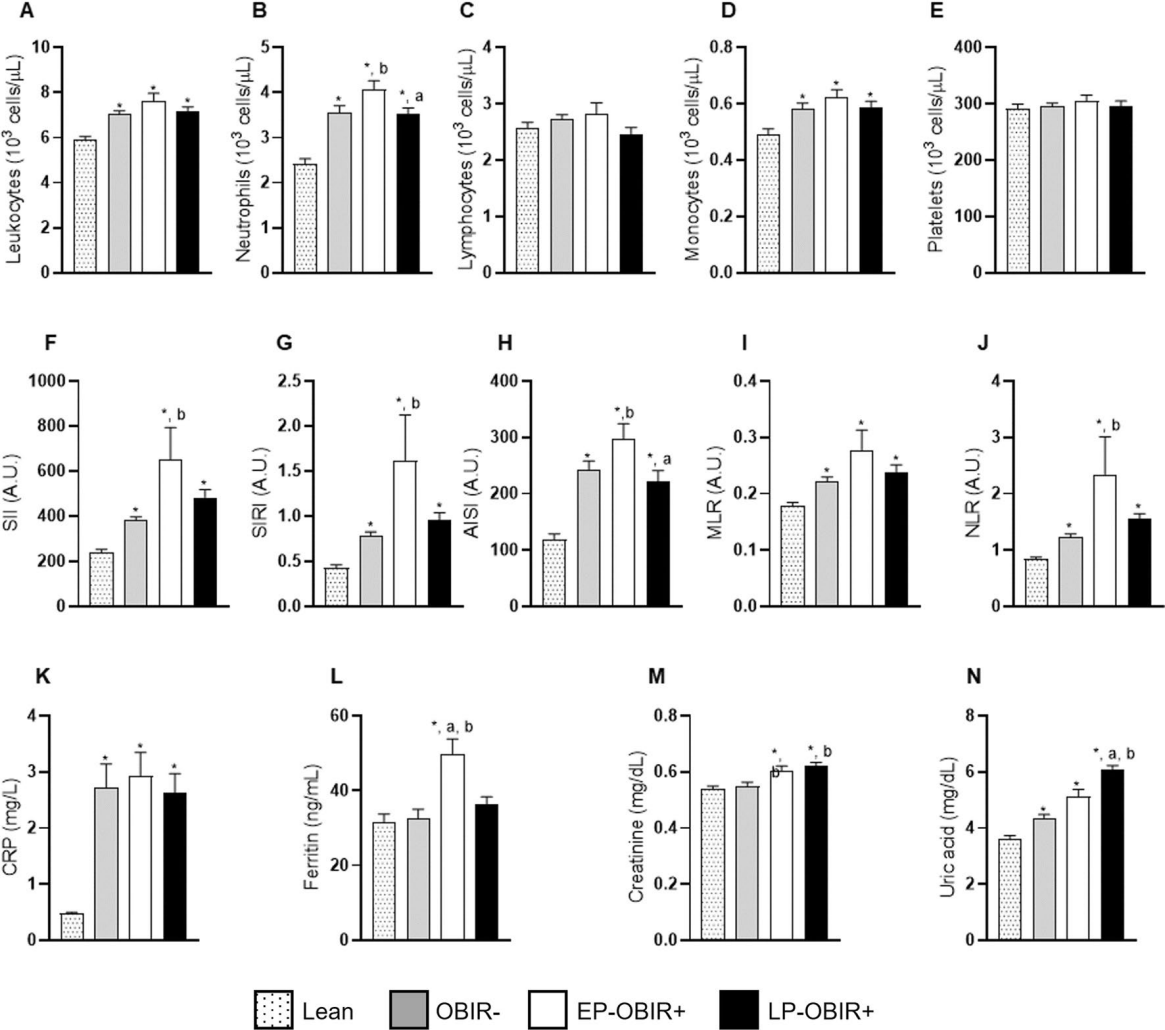
Inflammatory markers of the study population. Total leukocyte count **A**, total neutrophil count **B**, total lymphocyte count **C**, total monocyte count **D**, total platelet count **E**, systemic immune-inflammation index **F**, systemic inflammation response index **G**, aggregate index of systemic inflammation **H**, monocyte to lymphocyte ratio **I**, neutrophil to lymphocyte ratio **J**, plasmatic C-reactive protein **K**, plasmatic ferritin **L**, plasmatic creatinine **M**, and plasmatic uric acid **N**. White bars with dots represent lean children (n = 30), grey bars represent metabolically healthy children with obesity (n = 35), white bars represent the early responder group (n = 19), and black bars represent the late responder group (n = 32). Values are means ± SEM. P < 0.05 was considered for statistical significance. (*) shows significant differences relative to lean children, **a** shows significant differences relative to EP-OBIR + children, **b** shows significant differences relative to OBIR- children, and (#) shows significant differences along the OGTT. ObIR-, non-insulin resistant children with obesity; ObIR + , insulin resistant children with obesity; EP-OBIR + , early peak OBIR + children; LP-OBIR + , late peak OBIR + children; SII, systemic immune-inflammation index; SIRI, systemic inflammation response index; AISI, aggregate index of systemic inflammation; MLR, monocyte to lymphocyte ratio; NLR, neutrophil to lymphocyte ratio; A.U., arbitrary units; CRP, C-Reactive Protein

All three groups of children with obesity had increased leucocyte counts, explained by increased total neutrophils and monocytes, while lymphocytes were similar in all four groups. Furthermore, the EP-OBIR + group had more neutrophils than any other group. In general terms, LP-OBIR + children tended towards lower WBC counts when compared to EP-OBIR + children (Fig. 5A–D). Total platelet count did not vary between groups either (Fig. 5E), hence the inflammatory ratios derived from these were increased in every group with obesity when compared with controls, but the EP-OBIR + group presented higher levels than any of the other groups (Fig. 5F–J). Regarding plasmatic inflammation markers, again, every group with obesity showed similarly increased levels of CPR when compared to controls, while the EP-OBIR + group had higher ferritin levels than the rest. The LP-OBIR + group had the highest uric acid levels, and both OBIR + groups had higher creatinine levels than the others (Fig. 5K, N).

### Inflammasome activation

**Fig. 6:**
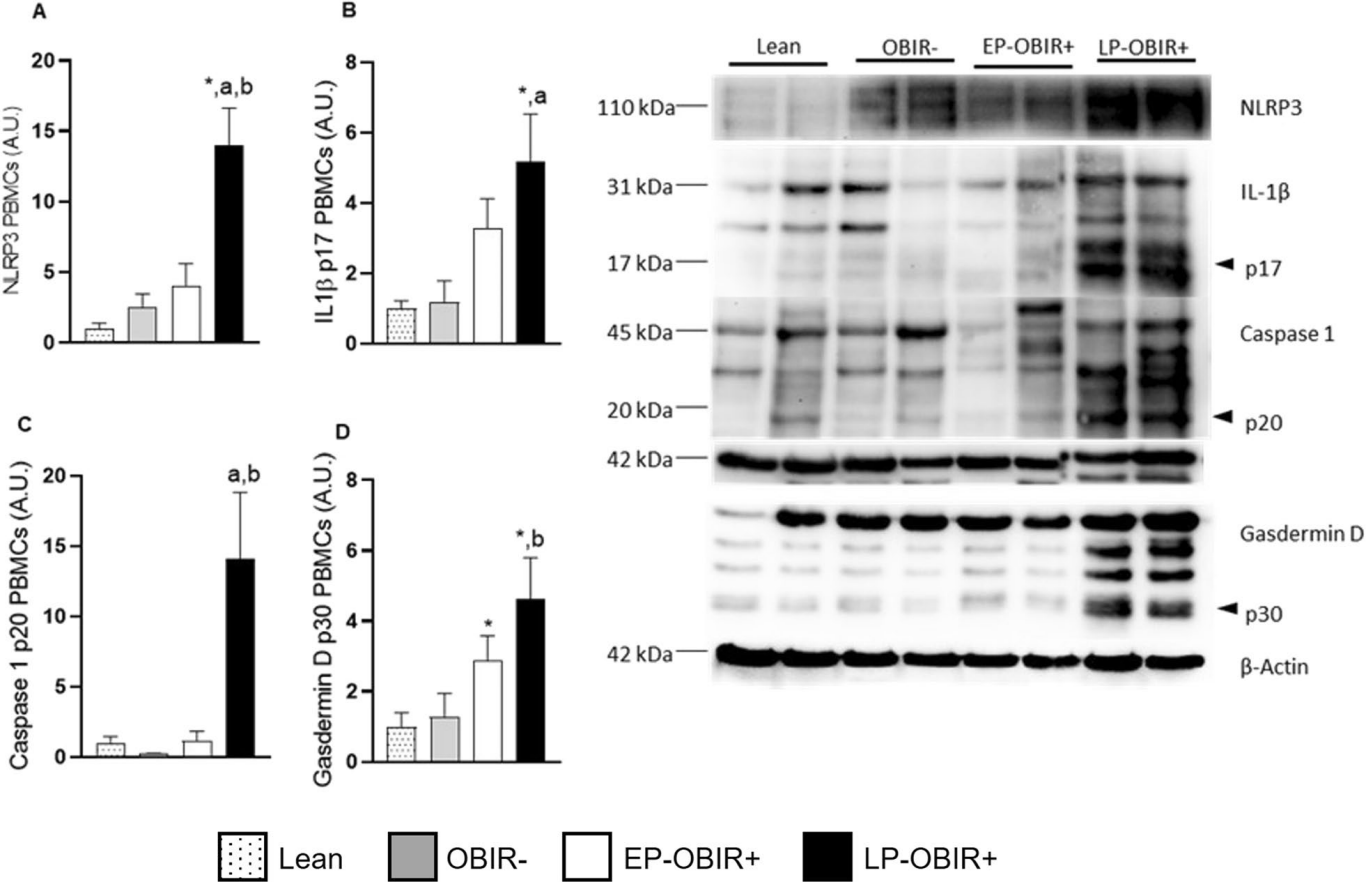
NLRP3 **A**, IL-1β **B**, caspase 1 **C**, and gasdermin **D** were quantified by western blotting in PBMCs of the study population. White bars with dots represent lean children (n = 8), grey bars represent OBIR- children (n = 8), white bars represent EP-OBIR + children (n = 12), and black bars represent LP-OBIR + children (n = 14). Values are means ± SEM. P < 0.05 was considered for statistical significance. The panel on the right are representative images of the western-blots performed for quantification. (*) shows significant differences relative to lean children, **a** shows significant differences relative to EP-OBIR + children, and **b** shows significant differences relative to OBIR- children. A.U., arbitrary units; NLRP3, NOD like receptor 3; IL-1β, interleukin 1β; PBMCs, peripheral blood mononuclear cells; ObIR-, non-insulin resistant children with obesity; ObIR + , insulin resistant children with obesity; EP-OBIR + , early peak OBIR + children; LP-OBIR + , late peak OBIR + children

The LP-OBIR + group exhibited markedly higher levels of PBMCs cellular NLRP3 and its effector proteins (active IL-1β, caspase, 1 or gasdermin D). In the case of gasdermin D, the EP-OBIR + group also had higher levels than controls and OBIR- children, but still, the LP-OBIR + group showed a further increase (Fig. 6A–D).

**Fig. 7:**
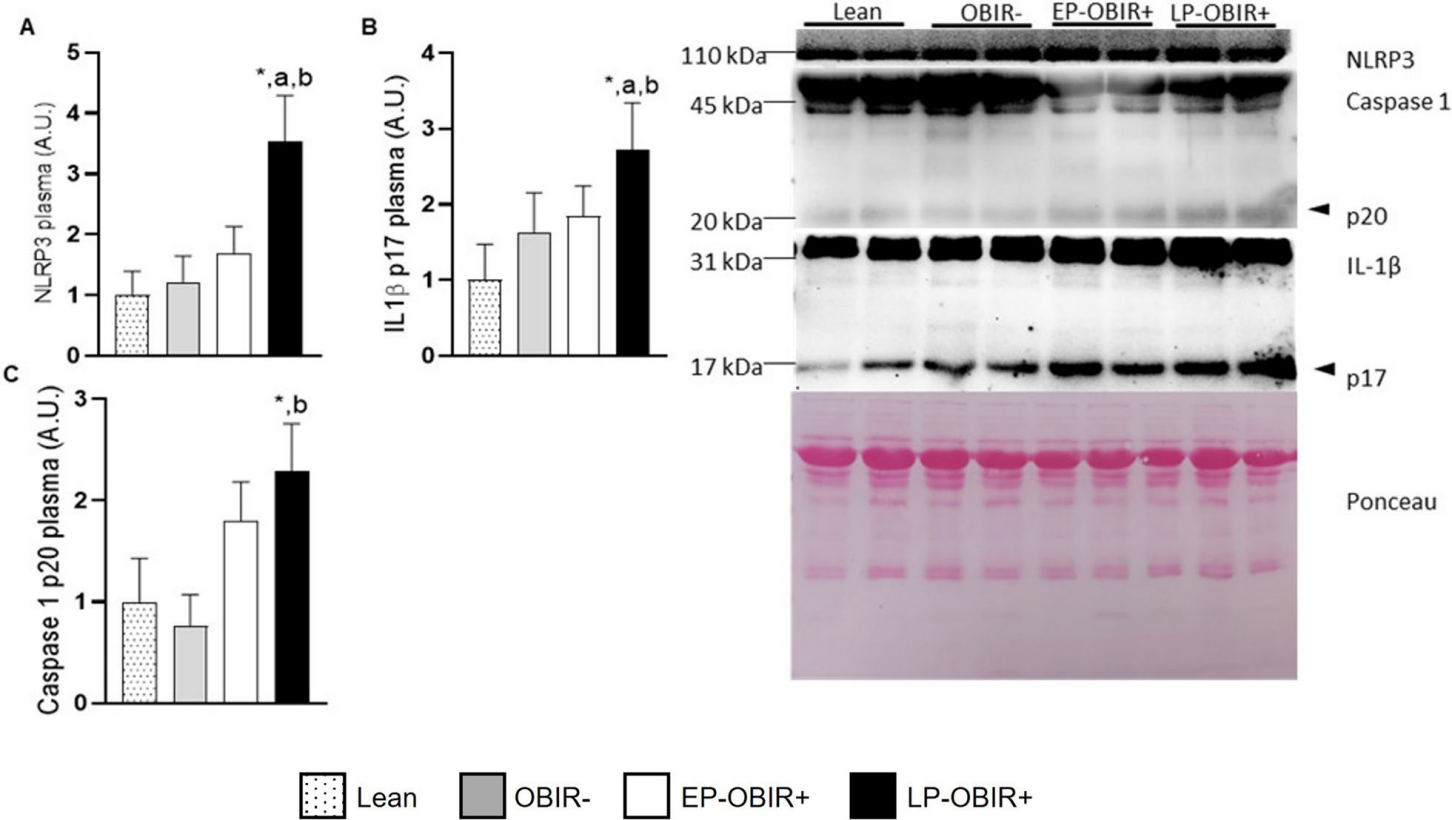
NLRP3 **A**, IL-1β **B**, and caspase 1 **C** were quantified by western blotting in plasma of the study population. White bars with dots represent lean children (n = 8), grey bars represent OBIR- children (n = 8), white bars represent EP-OBIR + children (n = 12), and black bars represent LP-OBIR + children (n = 14). Values are means ± SEM. P < 0.05 was considered for statistical significance. The panel on the right are representative images of the western-blots performed for quantification. (*) shows significant differences relative to lean children, **a** shows significant differences relative to EP-OBIR + children, and **b** shows significant differences relative to OBIR- children. A.U., arbitrary units; NLRP3, NOD like receptor 3; IL-1β, interleukin 1β; PBMCs, peripheral blood mononuclear cells; ObIR-, non-insulin resistant children with obesity; ObIR + , insulin resistant children with obesity; EP-OBIR + , early peak OBIR + children; LP-OBIR + , late peak OBIR + children

Circulating NLRP3 and IL-1β were also increased in the plasma of LP-OBIR + individuals relative to the other study groups, while circulating caspase 1 was only found to be increased in the LP-OBIR + group relative to controls and the OBIR- group (Fig. 7A–C). Moreover, as shown in Additional file 1: Fig. S4, some of the inflammasome components showed great diagnostic potential as reflected by the obtained areas under the curve for their respective ROC curves (0.84 and 0.78 for NLRP3 and caspase 1 in PBMCs, respectively, and 0.72 and 0.79 for NLRP3 and IL-1β in plasma, respectively).

### Correlation analysis

**Fig. 8:**
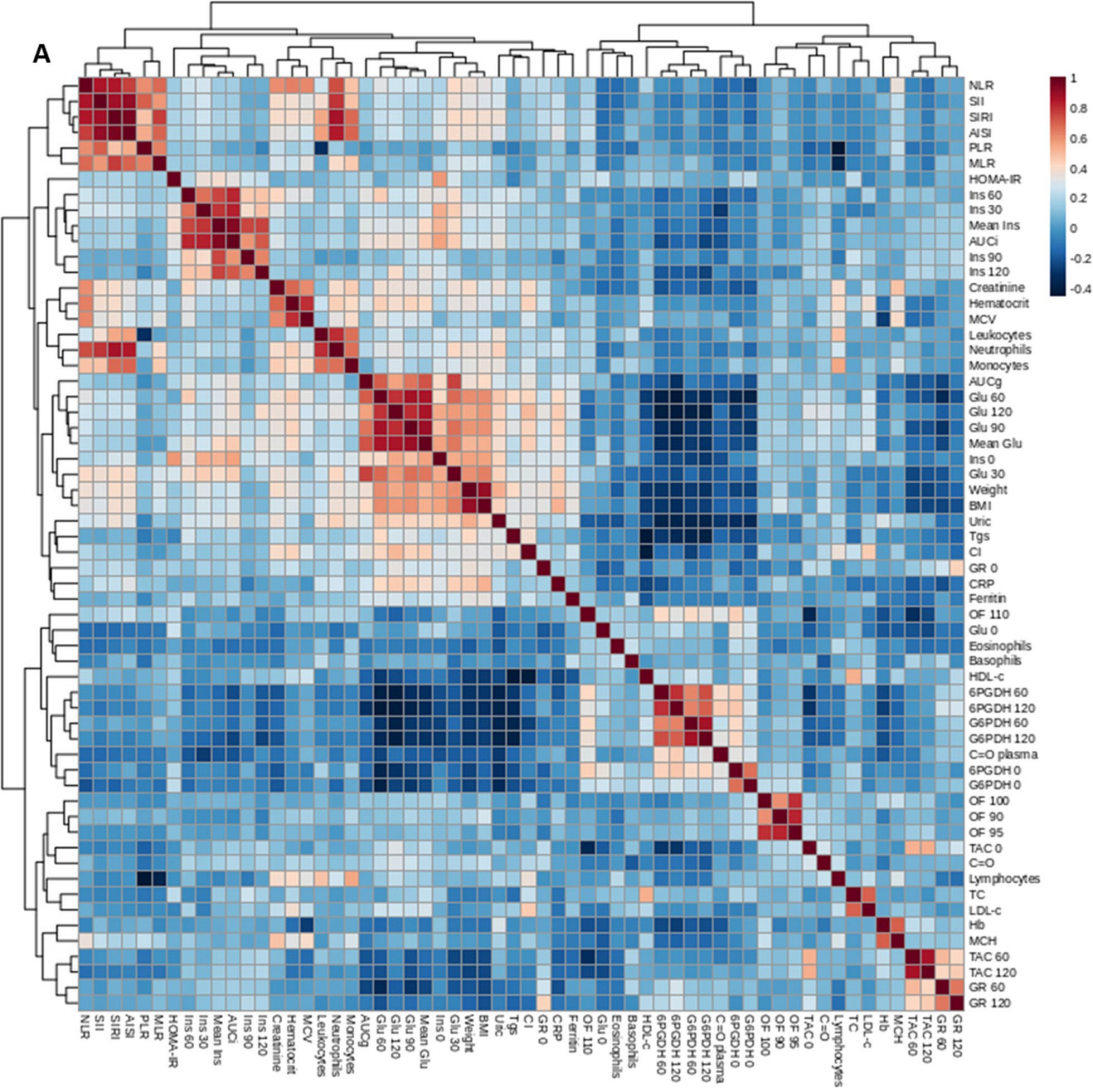

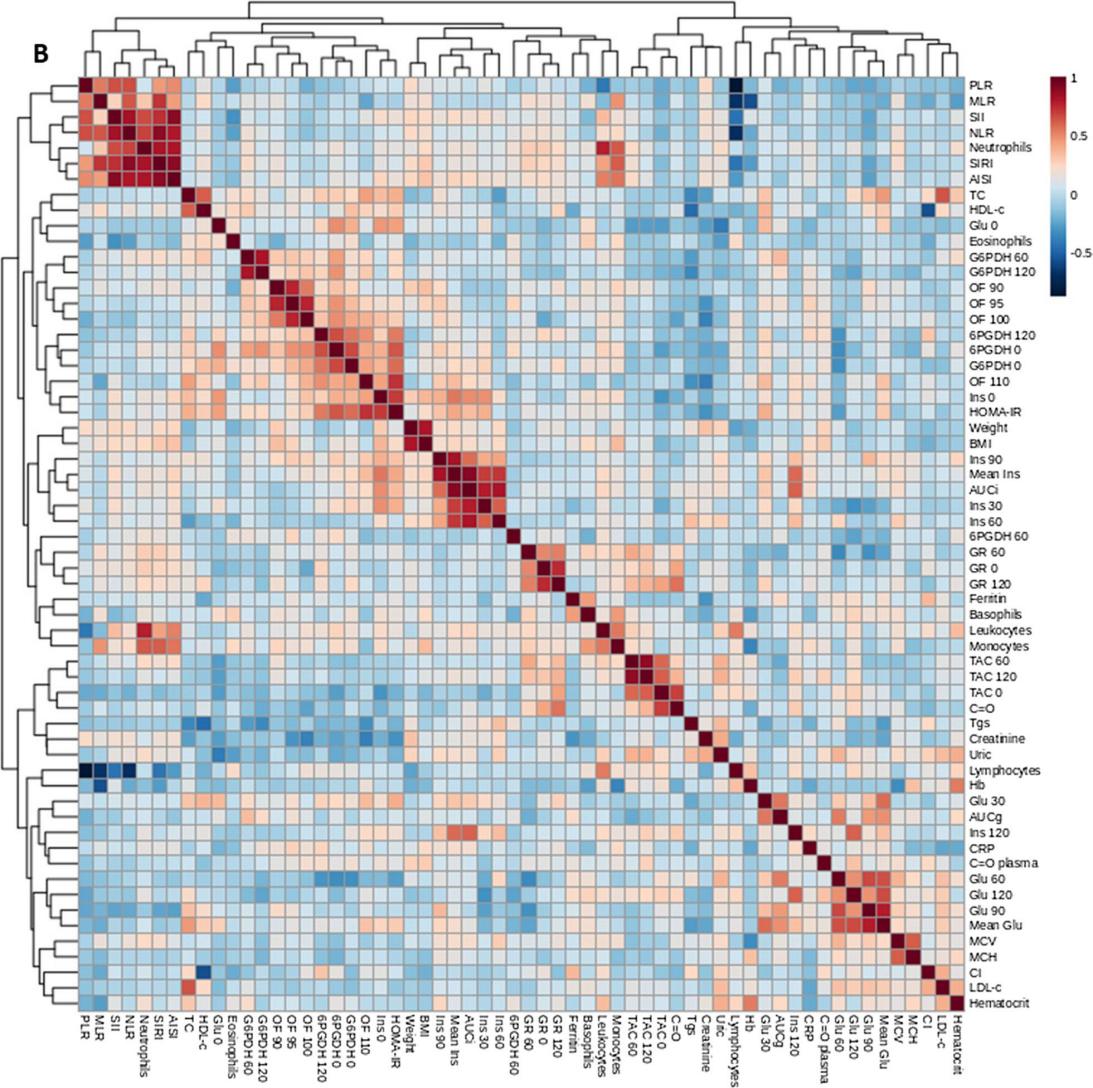
Representation of Spearman’s correlations between anthropometric and biochemical variables in every group under study **A** and in in metabolically unhealthy children with obesity **B**. P < 0.05 was considered for statistical significance. CI, Castelli index; SII, systemic immune-inflammation index; SIRI, systemic inflammation response index; AISI, aggregate index of systemic inflammation; NLR, neutrophil to lymphocyte ratio; MLR, monocyte to lymphocyte ratio; Ins, insulin; Glu, glucose; 6PGDH, 6-phosphogluconate dehydrogenase; G6PDH, glucose-6-phosphate dehydrogenase; GR, glutathione reductase; TC, total cholesterol; Tgs, triglycerides; C = O, carbonyl groups; BMI, body mass index; TAC, total antioxidant capacity; CRP, C-reactive protein; Hb, hemoglobin; OF, osmotic fragility; MCV msupplean corpuscular volume; MCH, mean corpuscular hemoglobin; HOMA-IR, homeostasis model assessment of insulin resistance; AUCg, area under the curve of glucose; AUCi, area under the curve of insulin; HDL-C, high density lipoprotein cholesterol; LDL-C, low density lipoprotein cholesterol

The correlation analysis including all groups showed obesity and carbohydrate metabolism-related parameters to be directly related to inflammation-related parameters, both canonical and WBC derived parameters. This was as expected for lipid metabolism parameters, with LDL-C, triglycerides, and Castelli index showing direct correlations with the mentioned proinflammatory markers, and HDL-C showing inverse correlations. WBC also correlated with canonical proinflammatory markers in plasma. Respect oxidative status WHAT IS THIS?, both G6PDH and 6PGDH showed negative correlations with obesity and carbohydrate metabolism-related parameters, as well as with inflammatory markers, whilst the behavior was the opposite for C = O and baseline GR activity (Fig. 8A).

Considering only OBIR + children, correlation analyses pointed again to a relation between obesity and carbohydrate metabolism-related parameters and inflammation-related parameters. Additionally, high baseline insulin and glucose levels correlated with higher dehydrogenase activity, whilst insulin and glucose at the final points of the OGTT showed the opposite behavior, and a direct correlation with C = O. Again, HDL-C showed a direct relationship with G6PDH activity. Also, high WBC counts were related to a better antioxidant defense. Finally, uric acid directly correlated with an inferior lipid metabolism, as well as with glucose levels along the OGTT (Fig. 8B).

## Discussion

Childhood obesity and MS are pathophysiologycally associated with inflammation and OS, which are in turn highly interrelated and potentiate each other. Indeed, children with MS exhibit depleted or ineffective cellular antioxidant defenses, together with a pro-oxidative metal homeostasis [28–31]. MS is known to be a risk factor for T2DM and cardiovascular disease. IR has plays a pivotal role in the development of these metabolic complications. However, we still do not have a definition of IR in children, and following ADA’s recommendations, increased basal insulin or a high insulin peak anywhere on the OGTT will serve as IR diagnostic criteria. A depletion in rapid insulin secretion is related to T2DM [4, 5] and worse metabolic profiles [6]. We aimed to study whether this altered insulin dynamics relates to OS and inflammation. As an experimental approach, we classified children with IR in two groups according to their insulin secretion dynamics, and OS and inflammatory parameters were assessed.

In line with recent findings, children with LP-OBIR + had a worse lipid profile [6] and higher glucose levels at the final phase of the OGTT, with higher mean glucose along the OGTT and increased ROS levels. High glucose may exert cytotoxic effects through ROS generation and this has been shown to affect the first phase of glucose-stimulated insulin secretion (GSIS) [32]. Glutathione enhances this GSIS in patients with impaired glucose tolerance [33]. Moreover, treating cellular and animal models of T2DM with antioxidant compounds results in the prevention of glucose-mediated reduction of insulin gene expression [34]. Animal models of T2DM have been described presenting higher glucokinase protein levels and activity [32]. Cellular models of increased glucokinase activity respond to glucose with enhanced glycolysis, decreased NADPH levels, oxidative damage, and cell death that is partially prevented if ROS are reduced with scavengers [32]. In accordance, we found LP-OBIR + children to be unable to respond to the stress of an acute intake of glucose by increasing TAC, GR and the main erythroid dehydrogenase activities (G6PDH and 6PGDH).

Indeed, both G6PDH and 6PGDH activities were inversely correlated with BMI, inflammation, and carbohydrate metabolism-related parameters. whilst C = O was directly correlated. This suggests a relation between antioxidant defenses and cellular metabolism. Furthermore, we found depleted GR activity in the LP-OBIR + group. GR is responsiblefor recycling reduced glutathione from its oxidized form, which is crucial for a correct antioxidant defense and the maintenance of the redox status. Such depletion matches with the previously described requirement for glutathione reduction for a correct first phase of GSIS [35]. Thus, NADPH and the pentose phosphate pathway, needed for reduced glutathione recycling, seem relevant regulators of GSIS as well. Indeed, G6PDH and 6PGDH inhibition leads to GSIS blockage, OS and β-cells apoptosis [36]. The LP-OBIR + group also presented increased levels of oxidative damage markers in red blood cells at baseline, and glucose intake resulted in the worsening of the oxidative status. On the other hand, the EP-OBIR + group behaved much like OBIR- children. In line with this increased erythroid oxidative damage, LP-OBIR + children also appeared to show increased osmotic fragility. Obesity has been related to higher Hb, red blood cell count and hematocrit [37], which is what we found in the late responder group. Hb and hematocrit are associated with higher risks of developing IR, T2DM and hyperuricemia [38–40]. LP-OBIR + children also exhibited higher levels of plasmatic TBARS and C = O at baseline and these may act as DAMPs, enhancing inflammatory responses in surrounding tissues [18].

Surprisingly, the LP-OBIR + group, did not show the most adverse inflammatory profile. In fact, it was the EP-OBIR + that displayed higher neutrophil counts, SII, SIRI, AISI, and NLR indexes and ferritin levels. Only uric acid was higher in the LP-OBIR + group than in the EP-OBIR + group, which, like OBIR-, had higher levels than controls. In obesity, uric acid accumulation is the result of the interplay between a complex set of factors, such as diet (e.g., consumption of fructose-rich foods is associated with metabolic complications, since fructose metabolism yields uric acid) or altered metabolism (increased production derived from, for example, increased cell turnover and decreased renal excretion of urate) (see Additional file 1: Fig. S5). Moreover, although in analyses with the overall population WBC counts correlated with plasmatic proinflammatory markers, within OBIR + children these parameters appeared to be related to a better antioxidant defense. Thus, it would seem as if a low-grade inflammation does not produce a worse metabolic signature in children. None of these differences could be predicted by any other parameter at baseline. Basal glucose, triglycerides, total cholesterol and subfractions, or Castelli index did not differ between any of the groups with obesity, and HOMA-IR, and basal insulin were higher in both OBIR + groups, without differences between them.

The inflammation markers and indices studied have been associated with higher BMI or obesity [41–43]. In line with this, obesity is accompanied by changes in hematologic counts such as increased macrophage and monocyte tissue infiltration, and higher total leukocyte, neutrophil and lymphocyte counts [44]. In contrast we found children in the LP-OBIR + group to present diminished neutrophil counts. In our cohort, WBC derived inflammatory indexes, as well as traditional inflammatory markers such as uric acid, directly correlated with altered erythrocyte physiology (hematocrit, hemoglobin, MCV, and MCH) and increased osmotic fragility, whilst the relationship was inverse between these erythroid parameters and dehydrogenases activities and TAC, respectively, suggesting a positive role of erythroid antioxidant defenses in the maintenance of red blood cell physiology. In OBIR + children, total hemoglobin levels inversely related to MLR. Finally, uric acid levels directly correlated with insulin levels 120 min after the glucose intake, in line with the fact that LP-OBIR + children present higher uric acid levels.

Hence, thus far our data show that the LP-OBIR + group suffer OS events, while the EP-OBIR + group display a worse inflammatory status according to traditional markers, except uric acid. Uric acid is a well-known trigger of NLRP3 priming. NLRP3 acts as an intracellular sensor of danger signals which, after assembly, mediates a process of caspase 1-dependent cytokine release through gasdermin-D activation. After gasdermin-D activation, a pore is formed in the inner cell membrane, so pyroptotic cell death and further cytokine release are accomplished [45]. PBMCs extracted from children of the LP-OBIR + group presented higher levels of NLRP3, IL-1β, caspase 1 and gasdermin-D proteins when compared to the other study groups. Probably, as an effect of gasdermin-D mediated pyroptotic PBMCs death, this resulted in higher levels of NLRP3 and IL-1β in plasma as well. Circulating levels of NLRP3 have been associated with MS components [46] and other pathologies in which it may serve as a predictive biomarker [47, 48]. This pathological mechanism could at least partially explain the reduced white blood cell counts in this group and therefore, the lower levels of inflammation markers in the LP-OBIR group. Moreover, circulating inflammasome components could represent a pathological endocrine mechanism of inflammatory response [49]. Accordingly, ex vivo PMBCs (from a set of healthy young adult volunteers) incubated with uric acid, resulted in reduced cell viability (Additional file 1: Fig. S3A), increased levels of cellular and free NLRP3 and IL-1β proteins until extremely cytotoxic uric acid concentrations were reached (Additional file 1: Figure S1). These harmful effects were reverted with the addition of ascorbic acid to the cultures (Additional file 1: Figs. S2 and S3B). Uric acid’s role in peripheral blood cells is controversial [50, 51]. Nevertheless, the role of uric acid in hepatic steatosis and IR through NLRP3 inflammasome activation has already been described [52], and compounds targeting serum uric acid levels have been proposed as therapeutic approaches for nonalcoholic fatty liver disease treatment [53]. Overnutrition induces β-cell dysfunction affecting insulin secretion through mitochondrial ROS generation and NLRP3 activation [54, 55]. NLRP3 pathway inhibition avoids adipose tissue inflammation and diminishes obesity and related metabolic disorders [49], and the use of natural compounds with antioxidant capacity, such as polyphenols or carotenoids, has been described as having beneficial effects in the control of diabetic complications through NLRP3 pathway control [56–60].

This data should be interpreted with caution, since it has been derived from an observational study with no follow-up. In this vein, it should be noted that the multifactorial nature of childhood obesity, along with the observational design of our study, makes it difficult to weigh the importance of each factor analyzed in the outcome. It would be desirable to perform long-term prospective studies to properly identify relative risks, and determine whether treatment with antioxidant-rich functional nutrition could reverse this situation despite weight excess.

In summary, we have found that not every child with obesity and IR has OS and deleterious inflammation, and that IR as we define it today [20], is not a precise marker of obesity related complications. Our data suggest that the appearance of altered prandial insulin secretion reflected in OGTT is a better indicator of increased inflammasome activation and OS damage and therefore, of a higher risk of the development of obesity-related metabolic complications. Finally, uric acid could be useful to identify children with obesity at higher risk of delayed insulin response, OS and inflammasome activation.

## Conclusions

Altered insulin secretion dynamics in response to glucose along an OGTT effectively identifies children with obesity suffering OS and inflammasome activation, despite similar basal glucose, insulin and lipid profiles as well as classical inflammatory markers. This should lead to a rethink of our actual definition of IR in children, drive the search for better and less invasive biomarkers, and to explore the potential benefits of anti-oxidants beyond weight loss.

### Supplementary Information

**Additional file 1: Figure S1.** Uric effect over PBMCs inflammasome components. PBMCs **A** and released to the medium **B** levels of NLRP3 and its final effector after incubation with different uric acid concentrations. PBMCs were either left untreated (CT-), treated with LPS and ATP (CT+) or treated with different uric acid concentrations (180-10.000 μM) and ATP. CT, control; NLRP3, NOD like receptor 3, IL-1β, interleukin 1β; PBMCs, peripheral blood mononuclear cells. **Figure S2.** Ascorbic acid effect over uric acid-induced inflammatory response. PBMCs **A** and released to the medium **B** levels of NLRP3 and its final effector after incubation with uric acid and different ascorbic acid concentrations. PBMCs were either left untreated (CT-), treated with LPS and ATP (CT+) or treated with uric acid 400 μM and ATP in medium supplemented with different ascorbic acid concentrations (0-10.000 μM). CT, control; NLRP3, NOD like receptor 3, IL-1β, interleukin 1β; PBMCs, peripheral blood mononuclear cells; LPS, lipopolysaccharide; ATP, adenosine triphosphate. **Figure S3.** Cellular viability of PBMCs incubated with different uric acid concentrations (0-5000 μM) and ATP **A**, and with uric acid 400 μM and ATP under supplementation with different ascorbic acid concentrations (500-10000 μM) **B**. Values are means ± SEM. P<0.05 was considered for statistical significance. (*) shows significant differences relative to negative controls. CT, control; PBMCs, peripheral blood mononuclear cells; LPS, lipopolysaccharide; ATP, adenosine triphosphate. **Figure S4.** ROC curves for NLRP3 in PBMCs and plasma **A**, **E**, IL-1 β in PBMCs and plasma **B**, **F**, caspase-1 in PBMCs and plasma **C**, **G**, and gasdermin **D** in PBMCs **D**. **Figure S5.** Metabolic alterations described in obesity leading to uric acid accumulation. ATP, adenosine triphosphate; AMP, adenosine monophosphate; IMP, inosine monophosphate; XMP, xanthosine monophosphate; GMP, guanosine monophosphate; URAT1, urate-anion transporter 1; ABCG2, ATP binding cassette subfamily G member 2.

## Data Availability

Data are available upon request. Data shared are in accordance with consent provided by participants on the use of confidential data.

## Abbreviations

OGTT: Oral glucose tolerance
DAMPs: Danger-associated molecular patterns
MS: Metabolic syndrome
IR: Insulin resistance
OS: Oxidative stress
ObIR-: Non-insulin resistant children with obesity
ObIR +: Insulin resistant children with obesity
EP-OBIR +: Early peak OBIR + children
LP-OBIR +: Late peak OBIR + children
C = O: Carbonyl groups
GR: Glutathione reductase
