# Supplementary material for: Altered insulin secretion dynamics relate to oxidative stress and inflammasome activation in children with obesity and insulin resistance
Source: J Transl Med. 2023 Aug 20;21:559. doi: 10.1186/s12967-023-04337-7 (PMC10440893; doi:10.1186/s12967-023-04337-7)
Supplement: Supplementary file 1 — Additional file 1: Figure S1. Uric effect over PBMCs inflammasome components. PBMCs A and released to the medium B levels of NLRP3 and its final effector after incubation with different uric acid concentrations. PBMCs were either left untreated (CT-), treated with LPS and ATP (CT+) or treated with different uric acid concentrations (180-10.000 μM) and ATP. CT, control; NLRP3, NOD like receptor 3, IL-1β, interleukin 1β; PBMCs, peripheral blood mononuclear cells. Figure S2. Ascorbic acid effect over uric acid-induced inflammatory response. PBMCs A and released to the medium B levels of NLRP3 and its final effector after incubation with uric acid and different ascorbic acid concentrations. PBMCs were either left untreated (CT-), treated with LPS and ATP (CT+) or treated with uric acid 400 μM and ATP in medium supplemented with different ascorbic acid concentrations (0-10.000 μM). CT, control; NLRP3, NOD like receptor 3, IL-1β, interleukin 1β; PBMCs, peripheral blood mononuclear cells; LPS, lipopolysaccharide; ATP, adenosine triphosphate. Figure S3. Cellular viability of PBMCs incubated with different uric acid concentrations (0-5000 μM) and ATP A, and with uric acid 400 μM and ATP under supplementation with different ascorbic acid concentrations (500-10000 μM) B. Values are means ± SEM. P<0.05 was considered for statistical significance. (*) shows significant differences relative to negative controls. CT, control; PBMCs, peripheral blood mononuclear cells; LPS, lipopolysaccharide; ATP, adenosine triphosphate. Figure S4. ROC curves for NLRP3 in PBMCs and plasma A, E, IL-1 β in PBMCs and plasma B, F, caspase-1 in PBMCs and plasma C, G, and gasdermin D in PBMCs D. Figure S5. Metabolic alterations described in obesity leading to uric acid accumulation. ATP, adenosine triphosphate; AMP, adenosine monophosphate; IMP, inosine monophosphate; XMP, xanthosine monophosphate; GMP, guanosine monophosphate; URAT1, urate-anion transporter 1; ABCG2, ATP binding cassette subfamily G me [file 12967_2023_4337_MOESM1_ESM.docx]

Title: **Altered Insulin Secretion Dynamics Relate to Oxidative Stress and Inflammasome Activation in Children with Obesity and Insulin Resistance**

Authors:

Álvaro González-Domínguez^1^, Thalía Belmonte^2,3^, Jesús Domínguez-Riscart^1,4^, Pablo Ruiz-Ocaña^1,5^, Inés Muela-Zarzuela^6^, Ana Saez-Benito^7,8^, Raúl Montañez-Martínez^9^, Rosa M. Mateos^1,10^, and Alfonso M. Lechuga-Sancho^1,4,11*^

Institutions:

1. Grupo de Inflamación, Nutrición, Metabolismo y estrés Oxidativo. Instituto de Investigación e Innovación Biomédica de Cádiz (INiBICA). Cádiz. Spain.

2. Translational Research in Respiratory Medicine, University Hospital Arnau de Vilanova and Santa Maria, IRBLleida, Lleida, Spain.

3. CIBER of Respiratory Diseases (CIBERES), Institute of Health Carlos III, Madrid, Spain.

4. Unidad de Endocrinología Pediátrica y Diabetes. Servicio de Pediatría. Hospital Universitario Puerta del Mar, Cádiz, Spain

5. Unidad de Endocrinología Pediátrica y Diabetes. Servicio de Pediatría. Hospital Universitario de Jerez, Jerez de la Frontera, Spain.

6. Grupo de Inflamación y Metabolismo Durante el Envejecimiento. Instituto de Investigación e Innovación Biomédica de Cádiz (INiBICA). Cádiz. Spain.

7. Servicio de Análisis Clínicos. Hospital Universitario Puerta del Mar, Cádiz, Spain.

8. Grupo de Diabetes Mellitus - Autoinmunidad y complicaciones crónicas. Implicaciones Patológicas, clínicas y terapéuticas. Instituto de Investigación e Innovación Biomédica de Cádiz (INiBICA). Cádiz. Spain.

9. Grupo de Daño cerebral Perinatal. Instituto de Investigación e Innovación Biomédica de Cádiz (INiBICA). Cádiz. Spain.

10. Departamento de Biomedicina, Biotecnología y Salud Pública y Salud Pública, Facultad de Ciencias, Universidad de Cádiz, Puerto Real, Spain.

11. Departamento Materno Infantil y Radiología, Facultad de Medicina, Universidad de Cádiz, Cádiz, Spain.

* Correspondence should be addressed to:

Alfonso M. Lechuga-Sancho

Dept. Materno Infantil y Radiología

Universidad de Cádiz

Instituto de Investigación e Innovación en Ciencias Biomédicas (INiBICA)

c/ Doctor Marañón, 3 - Edificio Andrés Segovia

11002 Cádiz

Spain

email: alfonso.lechuga@uca.es

Running title: Inflammasome activation with Late Insulin Peak

**Supplementary Methods:**

*Diagnostic performance*

The diagnostic performance of inflammasome components was assessed performing receiver operating characteristic (ROC) curve analysis. An area under the curve (AUC) of 0.5 was considered to indicate no discrimination between groups, and an AUC of 1.0 a perfect discrimination. ROC curves were generated using GraphPad Prism 8 by plotting sensitivity against 1-specificity. The data was presented as the AUC and 95% confidence interval.

*PBMCs culture*

Isolated PBMCs were resuspended in RPMI medium (Fisher Scientific, Leicestershire, UK) and left at 37 ºC and 5% CO_2_ for 1 hour, allowing cellular acclimatation to the medium. Then, cells were transferred to p24 plates ensuring a minimum of 2000 cells per well and either left untreated (negative control), treated with LPS 6.7 ng/μL (positive control), or treated with uric acid at different concentrations (180-10.000 μM) for NLRP3 priming (Sigma-Aldrich, Saint Louis, USA). To test the effect of antioxidant supplementation over inflammasome activation, ascorbic acid was also added to uric acid wells at different concentrations (500-10000 μM). After 4 hours of incubation, adenosine triphosphate (ATP) 4.5 μM (Sigma-Aldrich, Saint Louis, USA) was added to every well (except negative control) to complete NLRP3 activation in another 30 minutes incubation step. Finally, medium was collected for protein precipitation in cold acetone (after centrifugation at 9600 g and 4 ºC) and cellular extracts for western blotting prepared as mentioned above.

*PBMCs viability assay*

Cell viability was assessed by 3-(4,5-dimethylthiazol-2-yl)-2,5-diphenyltetrazolium bromide (MTT) assay. Briefly, after PBMCs treatment, MTT is added to the wells at a final concentration of 500 μg/mL and incubated for 3 hours at 37 ºC and 5% CO_2_ to allow formazan crystals formation. Then, plates are centrifuged at 1600 g for 10 minutes, medium is discarded, and formazan is dissolved in a proper organic solvent, such as isopropanol with HCl 0.1 N. Finally, absorbance is registered at 595 nm.

On the other hand, and because of undesirable reactions between ascorbic acid and MTT ^1^, a potential protective role of antioxidant agents over PBMCs viability was assayed by counting of trypan blue stained cells. Briefly, 10 μL of cell suspension is mixed with 10 μL of trypan blue and cell viability is determined using an automatic cell counter (Biorad, Hercules, CA, USA).

**Supplementary figures.**

**
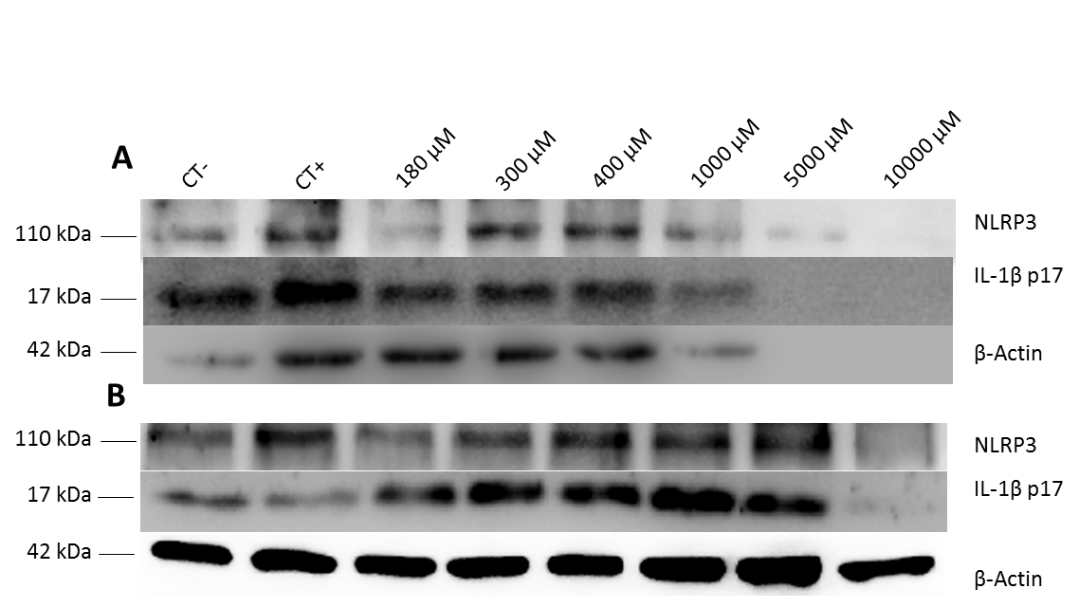
**

**Supplementary Figure 1.** Uric effect over PBMCs inflammasome components. PBMCs (A) and released to the medium (B) levels of NLRP3 and its final effector after incubation with different uric acid concentrations. PBMCs were either left untreated (CT-), treated with LPS and ATP (CT+) or treated with different uric acid concentrations (180-10.000 μM) and ATP. CT, control; NLRP3, NOD like receptor 3, IL-1β, interleukin 1β; PBMCs, peripheral blood mononuclear cells.


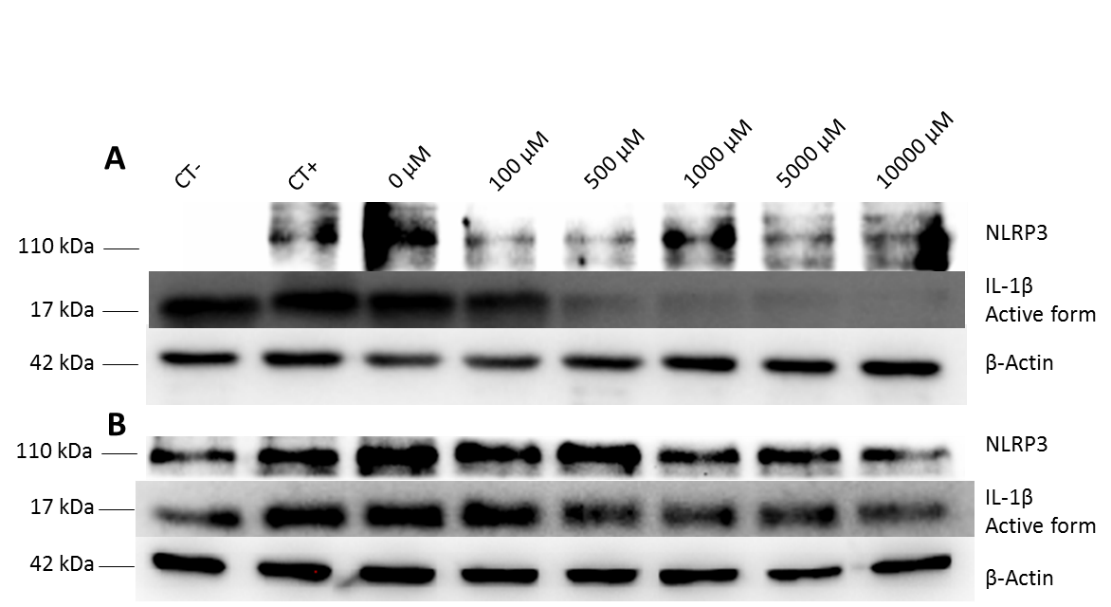


**Supplementary Figure 2.** Ascorbic acid effect over uric acid-induced inflammatory response. PBMCs (A) and released to the medium (B) levels of NLRP3 and its final effector after incubation with uric acid and different ascorbic acid concentrations. PBMCs were either left untreated (CT-), treated with LPS and ATP (CT+) or treated with uric acid 400 μM and ATP in medium supplemented with different ascorbic acid concentrations (0-10.000 μM). CT, control; NLRP3, NOD like receptor 3, IL-1β, interleukin 1β; PBMCs, peripheral blood mononuclear cells; LPS, lipopolysaccharide; ATP, adenosine triphosphate.

**
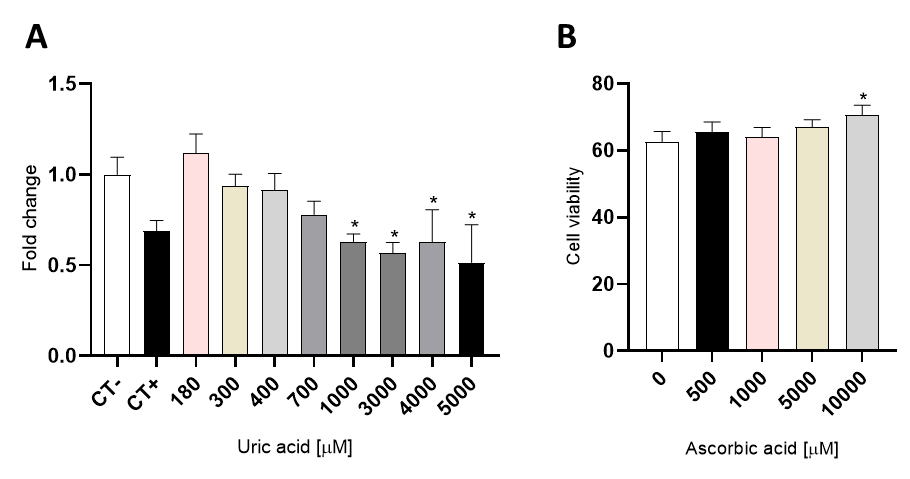
**

**Supplementary Figure 3.** Cellular viability of PBMCs incubated with different uric acid concentrations (0-5000 μM) and ATP (A), and with uric acid 400 μM and ATP under supplementation with different ascorbic acid concentrations (500-10000 μM) (B). Values are means ± SEM. P<0.05 was considered for statistical significance. (*) shows significant differences relative to negative controls. CT, control; PBMCs, peripheral blood mononuclear cells; LPS, lipopolysaccharide; ATP, adenosine triphosphate.


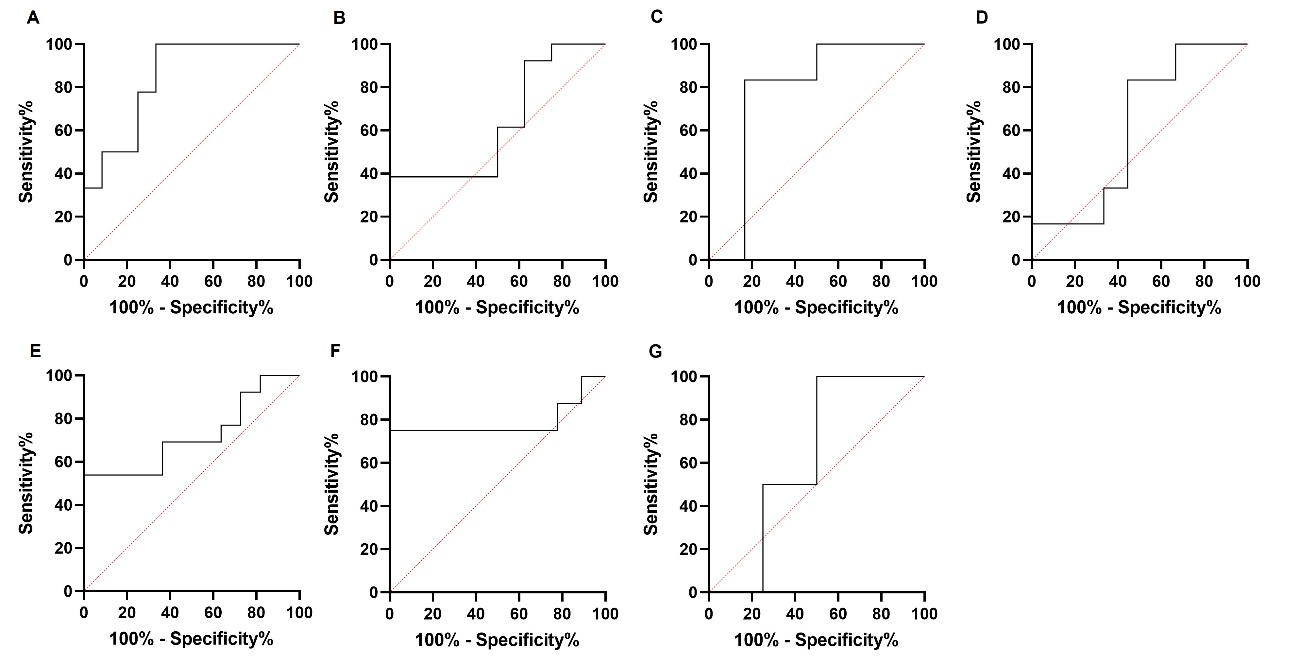


**Supplementary Figure 4.** ROC curves for NLRP3 in PBMCs and plasma (A,E), IL-1 β in PBMCs and plasma (B,F), caspase-1 in PBMCs and plasma (C,G), and gasdermin D in PBMCs (D).


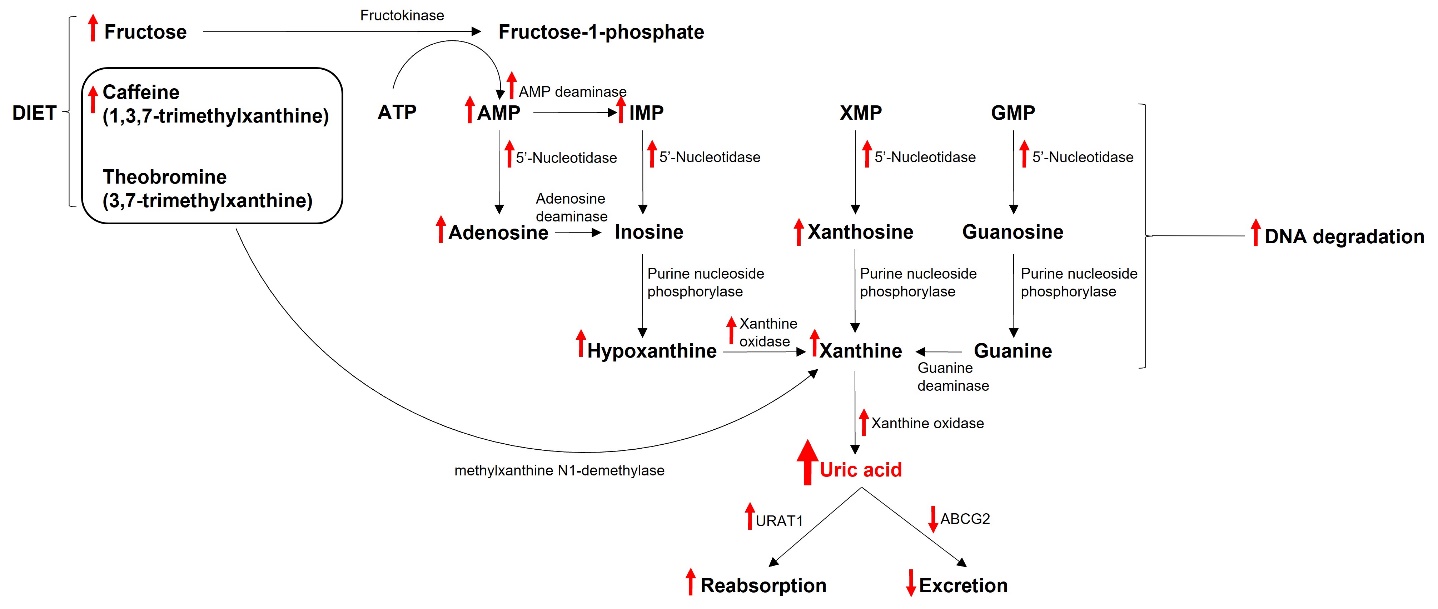


**Supplementary Figure 5.** Metabolic alterations described in obesity leading to uric acid accumulation. ATP, adenosine triphosphate; AMP, adenosine monophosphate; IMP, inosine monophosphate; XMP, xanthosine monophosphate; GMP, guanosine monophosphate; URAT1, urate-anion transporter 1; ABCG2, ATP binding cassette subfamily G member 2.

**Supplementary references.**

1. Natarajan M, Mohan S, Martinez B, Meltz M, Herman T. Antioxidant compounds interfere with the 3-[4,5-dimethylthiazol-2-yl]-2,5-diphenyltetrazolium bromide cytotoxicity assay. Cancer Detect Prev. 2000 Feb 1;24:405–14.

2. Li F, Chen S, Qiu X, Wu J, Tan M, Wang M. Serum Uric Acid Levels and Metabolic Indices in an Obese Population: A Cross-Sectional Study. Diabetes Metab Syndr Obes. 2021 Feb;Volume 14:627–35.

3. Czerwonogrodzka-Senczyna A, Rumińska M, Majcher A, Credo D, Jeznach-Steinhagen A, Pyrżak B. Fructose Consumption and Lipid Metabolism in Obese Children and Adolescents. In 2019. p. 91–100.

4. McCormick DP, Reyna L, Reifsnider E. Calories, Caffeine and the Onset of Obesity in Young Children. Acad Pediatr. 2020 Aug;20(6):801–8.

5. Jadhav AA, Jain A. Elevated adenosine deaminase activity in overweightand obese Indian subjects. Arch Physiol Biochem. 2012 Feb 23;118(1):1–5.

6. Perng W, Rifas-Shiman SL, McCulloch S, Chatzi L, Mantzoros C, Hivert MF, et al. Associations of cord blood metabolites with perinatal characteristics, newborn anthropometry, and cord blood hormones in project viva. Metabolism. 2017 Nov;76:11–22.

7. Stefanovic V, Antic S, Milojkovic M, Lazarević G, Vlahovic P. Lymphocyte ecto-5′-nucleotidase in obese type 2 diabetic patients treated with gliclazide. Diabetes Metab. 2006 Apr;32(2):166–70.

8. Tam HK, Kelly AS, Metzig AM, Steinberger J, Johnson LA. Xanthine Oxidase and Cardiovascular Risk in Obese Children. Childhood Obesity. 2014 Apr;10(2):175–80.

9. Escudero A, Carreño B, Retamal N, Celis C, Castro L, Aguayo C, et al. Elevated concentrations of plasma adenosine in obese children. BioFactors. 2012 Nov;38(6):422–8.

10. Dávila-Rodríguez MI, González-Salazar F, López-Cabanillas M, Cerda-Flores RM, Cortés-Gutiérrez EI. Evaluation of DNA damage in obese children using the chromatin dispersion test. Biotechnic & Histochemistry. 2023 May 23;1–5.

11. Doshi M, Takiue Y, Saito H, Hosoyamada M. The Increased Protein Level of URAT1 was Observed in Obesity/Metabolic Syndrome Model Mice. Nucleosides Nucleotides Nucleic Acids. 2011 Dec;30(12):1290–4.

12. Mishra J, Simonsen R, Kumar N. Intestinal breast cancer resistance protein (BCRP) requires Janus kinase 3 activity for drug efflux and barrier functions in obesity. Journal of Biological Chemistry. 2019 Nov;294(48):18337–48.
